# SMITH: spatially constrained stochastic model for simulation of intra-tumour heterogeneity

**DOI:** 10.1093/bioinformatics/btad102

**Published:** 2023-02-24

**Authors:** Adam Streck, Tom L Kaufmann, Roland F Schwarz

**Affiliations:** Max Delbrück Center for Molecular Medicine in the Helmholtz Association, Berlin Institute for Medical Systems Biology, Berlin, Germany; Institute for Computational Cancer Biology (ICCB), Center for Integrated Oncology (CIO), Cancer Research Center Cologne Essen (CCCE), Faculty of Medicine and University Hospital Cologne, University of Cologne, Cologne, Germany; BIFOLD—Berlin Institute for the Foundations of Learning and Data, Berlin, Germany; Institute for Computational Cancer Biology (ICCB), Center for Integrated Oncology (CIO), Cancer Research Center Cologne Essen (CCCE), Faculty of Medicine and University Hospital Cologne, University of Cologne, Cologne, Germany; Max Delbrück Center for Molecular Medicine in the Helmholtz Association, Berlin Institute for Medical Systems Biology, Berlin, Germany; Institute for Computational Cancer Biology (ICCB), Center for Integrated Oncology (CIO), Cancer Research Center Cologne Essen (CCCE), Faculty of Medicine and University Hospital Cologne, University of Cologne, Cologne, Germany; BIFOLD—Berlin Institute for the Foundations of Learning and Data, Berlin, Germany; Max Delbrück Center for Molecular Medicine in the Helmholtz Association, Berlin Institute for Medical Systems Biology, Berlin, Germany

## Abstract

**Motivation:**

Simulations of cancer evolution are highly useful to study the effects of selection and mutation rates on cellular fitness. However, most methods are either lattice-based and cannot simulate realistically sized tumours, or they omit spatial constraints and lack the clonal dynamics of real-world tumours.

**Results:**

Stochastic model of intra-tumour heterogeneity (SMITH) is an efficient and explainable model of cancer evolution that combines a branching process with a new confinement mechanism limiting clonal growth based on the size of the individual clones as well as the overall tumour population. We demonstrate how confinement is sufficient to induce the rich clonal dynamics observed in spatial models and cancer samples across tumour types, while allowing for a clear geometric interpretation and simulation of 1 billion cells within a few minutes on a desktop PC.

**Availability and implementation:**

SMITH is implemented in C# and freely available at https://bitbucket.org/schwarzlab/smith. For visualizations, we provide the accompanying Python package PyFish at https://bitbucket.org/schwarzlab/pyfish.

**Supplementary information:**

Supplementary data are available at *Bioinformatics* online.

## 1 Introduction

Carcinogenesis is governed by random mutational processes and selection, which give rise to intra-tumour heterogeneity (ITH), a main driver of progression, metastasis and treatment resistance (Marusyk *et al.*, 2020). To understand ITH, algorithms for inferring cancer evolution from, for example, sequencing data of clinical tumour specimens have proven highly valuable (Beerenwinkel *et al.*, 2015). Due to computational advances and the increasing availability of data, forward simulations of cancer evolution are now gaining traction (Noble *et al.*, 2022; West *et al.*, 2021), as they allow direct testing of biological hypotheses and modelling assumptions and enable the rapid exploration of the effects of fitness distributions and mutation rates on cancer evolution.

Models for simulating cancer evolution often employ variants of cellular automata, where cells or groups of cells are positioned on a 2D or a 3D lattice (Iwasaki and Innan, 2017). The lattice embedding directly creates spatial constraints which enable the simulation of, for example, biopsy results (Chkhaidze *et al.*, 2019) or the dispersal of cells in space (Waclaw *et al.*, 2015), or between neighbouring tissues (Noble *et al.*, 2022). However, capturing the mechanical behaviour of cells is difficult, and simplifying rules are frequently employed, for example, that a whole row of cells needs to be moved at once (Chkhaidze *et al.*, 2019), or that dead cells have to disappear from the lattice (West *et al.*, 2021). Additionally, simulating realistically sized tumours of 1–2 cm in diameter comprising around 1 billion cells (Del Monte, 2009) remains difficult even on supercomputer architecture (Rosenbauer *et al.*, 2020). Thus, cells are usually grouped to uniform populations of glands (Sottoriva *et al.*, 2015), demes (Noble *et al.*, 2022) or severely limited in number (West *et al.*, 2021).

Conversely, stochastic models of well-mixed populations, such as the commonly used branching process model of cancer (Haccou *et al.*, 2005), are highly scalable, but assume an exponentially growing population without spatial constraints. These unconstrained models only exhibit a limited amount of clonal dynamics and are characterized by a low number of driver mutations and low-to-medium clonal diversity (Noble *et al.*, 2022). They have been successfully applied to modelling clonal haematopoiesis, where space is not a primary limiting factor (Watson *et al.*, 2020), but their applicability to solid tumours remains limited. Therefore, there is a general need for efficient and scalable models of tumour evolution that include spatial constraints.

We therefore present Stochastic model of intra-tumour heterogeneity (SMITH)—a fast stochastic model of cancer evolution that can simulate realistically sized tumours of up to 1 billion cells. SMITH employs a classical branching model of cancer, with random cell birth and death processes modulated by fitness-increasing mutations, and without an explicit representation of cell location. SMITH additionally introduces *confinement*, a mechanic that limits the size of the growing population either by the size of the whole tumour (global confinement) or individually per clone (local confinement). Confinement separates the tumour into a proliferating shell and a static core, providing a natural way to represent both well-mixed and surface-growth models of cancer growth. Our approach is inspired by the observation that in solid tumours the number of cells that undergo cell division scales with the cell population raised to the power of two-thirds, corresponding to the surface area of a sphere (Talkington and Durrett, 2015).

We systematically assess the effects of confinement and demonstrate that increasing global confinement, that is, increasing overall selection pressure, causes an increase in the number of driver mutations in the simulated tumours, whereas increasing local confinement increases the clonal diversity. We show in principle and in comparison to real cancer data (Noble *et al.*, 2022) that local and global confinement are sufficient to recreate most commonly observed patterns of cancer evolution, including neutral, ‘linear’ and ‘branched’ evolution. Additionally, we leverage the high performance of SMITH to repeatedly simulate realistically sized tumour cell populations, orders of magnitude faster than explicit spatial models and evaluate the most important modelling assumptions and choices made in literature, such as population size and fitness distribution.

## 2 Materials and methods

Our model stochastically describes the size of tumour cell populations over time, thereby tracking the number of cells, their mutations and the evolutionary relationship between them. SMITH is based on a Galton–Watson branching process (Roch, 2015) and makes use of four key assumptions: (i) a low mutation probability with a non-negative fitness effect (driver mutations), (ii) a well-mixed population of cells, (iii) a spherical tumour shape and (iv) confined growth. Assumptions (i) and (ii) are common in branching process modelling and (iii) describes a well-studied group of tumours (Black and McGranahan, 2021). Assumption (iv) is specific to our model and directly changes the dynamics of the system. It is explained in detail in Section 2.5.

### 2.1 Model overview

A tumour as modelled by SMITH comprises two different types of cells: *alive* cells (which can be dividing or non-dividing) and *necrotic* cells. A newly created cell is always *alive*. Upon its death it can either be degraded (removed from the system) or remains embedded in the tumour as a *necrotic* cell, where it continues to contribute to the overall tumour mass, but does not divide any longer. SMITH does not model cellular position explicitly, it only keeps track of the number of cells in clones, where each clone is a collection of cells sharing the same set of mutations. Formally, a clone is a triplet $c=(c_{a},c_{n},c_{M})$, where $c_{a},c_{n}\in N_{0}$ describe the number of alive and necrotic cells in that clone, respectively, and *c_M_* describes the set of mutations shared by all the cells in *c*. Each mutation from the set of all possible mutations (m∈M) is unique. The set of possible mutations M is thereby considered infinite (infinite sites assumption; Kimura, 1969) and every mutation can only occur once.

SMITH is parametrized via $\theta =(\theta_{\text{mut}},\theta_{\text{fit}},\theta_{\text{conf}},\theta_{\text{local}})\in [0,1]\times R_{+}\times R_{+}\times R_{+}$, which describes the mutation probability per cell division, the average fitness increase of a mutation and the global and local confinement, respectively. Each parameter is explained in a corresponding section below.

To describe the state of the system over time $t\in N_{0}$, we denote as *C^t^* the set of clones at time step *t*. All simulations start from a single clone with a single alive cell characterized by a single identifying mutation, that is, $C^{0}=\{(1,0,\{m\})\}$. A simulation is then a transformation of *C^t^* into $C^{t+1}$ under the parameter set *θ*. For brevity, we use counts for alive and necrotic cells across the whole population as $C_{a}^{t}=\sum_{c^{t}\in C^{t}}c_{a}^{t},\;C_{d}^{t}=\sum_{c^{t}\in C^{t}}c_{d}^{t}$.

We use two stopping conditions for the simulation: the maximum number of time steps max_steps and the maximum population size max_pop. The simulation stops at a step *t* when $t=\text{max\_steps},\;(C_{a}^{t}+C_{n}^{t})>\text{max}\_\text{pop}$ or $C_{a}^{t}=0$. We also require the model to reach a minimum population min_pop. If the simulation terminates while $(C_{a}^{t}+C_{n}^{t})<\text{min}\_\text{pop}$ and t<max_steps, the result is discarded and the simulation restarts. For a schematic overview of the algorithm, see Supplementary Figure S1.

**Fig. 1.**
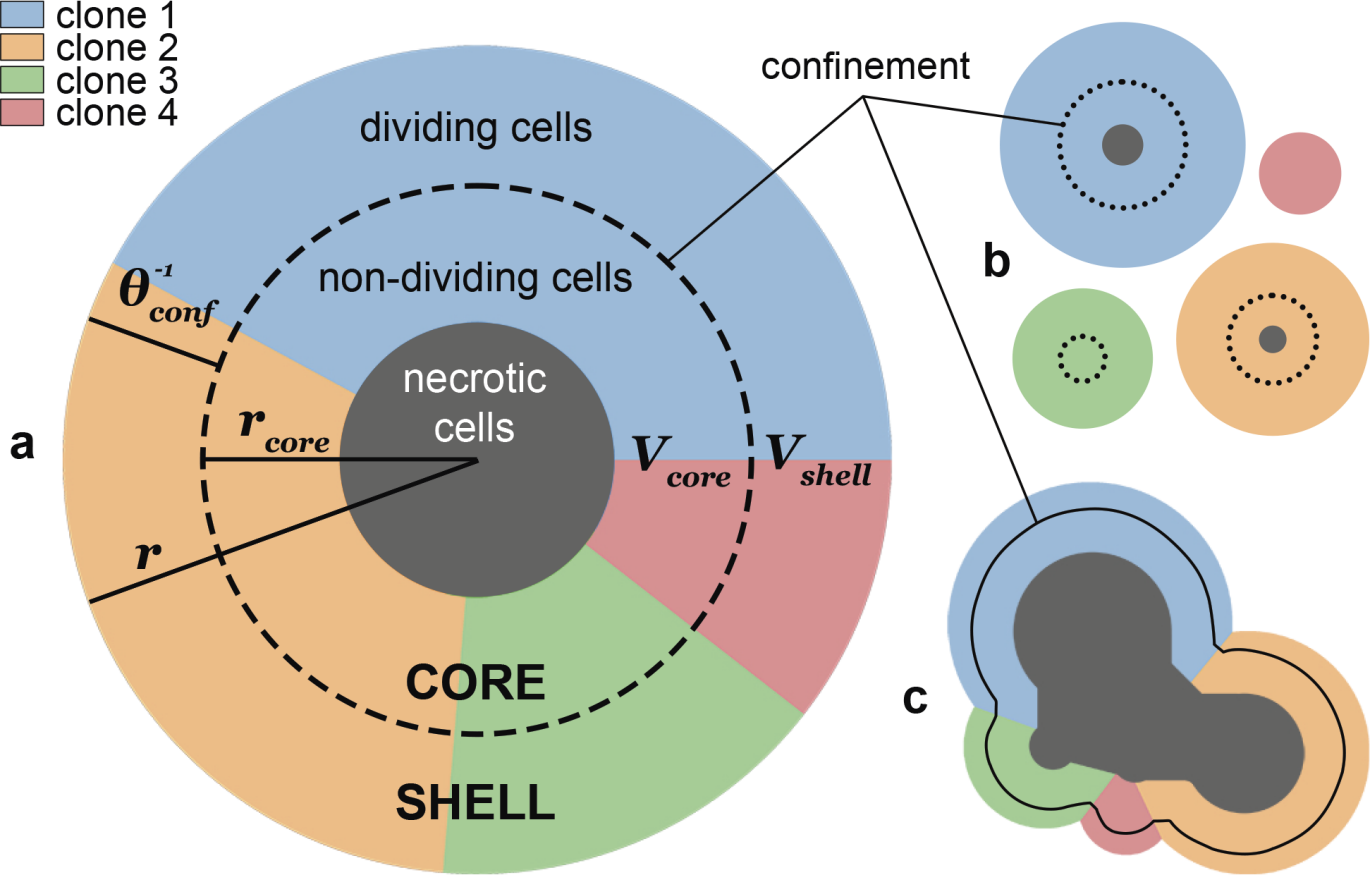
Confinement. (**a**) A tumour with four clones, where any individual cell is assumed to constitute one unit volume of space. Global confinement (dashed circle) splits the tumour into a proliferating shell, containing *V*_shell_ dividing cells, and a core with radius *r*_core_ of alive but not dividing and necrotic cells. The probability of a cell dividing is then a combination of its fitness and the probability of it being in the shell, given by the ratio $V_{\text{shell}}:(V_{\text{shell}}+V_{\text{core}})$. Similarly, dead cells are either removed or become necrotic based on the shell fraction. (**b**) A model with only local confinement (dotted circles). Each clone is independent and its growth is restricted only by its own population. (**c**) An approximate illustration of the combination of the global and the local confinement (solid outline). Clones compete for shared resources but are also limited by their own size

### 2.2 Cell turnover

We first assume a basic system in homeostasis where the number of cells is kept constant on average and without novel mutations or necrosis. We define a birth and death process by sampling the number of cells that are born $B_{b}(c^{t})$ and that have died $B_{d}(c^{t})$ at time step *t* in clone *c* from a binomial distribution. The granularity of each simulation step is given by the value step_size∈(0,1], with the birth probability function $p_{\text{birth}}(c^{t})=\text{step}\_\text{size}$ and the death probability $p_{\text{death}}(c^{t})=\text{step}\_\text{size}$. The number of alive cells at time step *t *+* *1 is then defined as: where $c^{t}\in C^{t}$ denotes the clone *c* at time step *t*. The next state of the simulation is then obtained as $C^{t+1}=\{c^{t+1}|c^{t}\in C^{t}\}$. Necrosis is introduced in Section 2.5, therefore in the homeostatic system $c_{n}^{t}$ stays at 0 for all *t*.


(1) $$B_{d}(c^{t})∼\text{Bin}(c_{a}^{t},p_{\text{death}}(c^{t})),$$

(2) $$B_{b}(c^{t})∼\text{Bin}(c_{a}^{t},p_{\text{birth}}(c^{t})),$$

(3) $$c_{a}^{t+1}=c_{a}^{t}+B_{b}(c^{t})-B_{d}(c^{t}),$$

Note that if $p_{\text{birth}}>p_{\text{death}}$, the population size increases exponentially. In the opposite case, the population would eventually die out. We thus set the birth and death probability equal to the turnover probability, keeping the population size constant on average. However, due to stochastic fluctuations, the population will always die out after a large, but finite number of steps (extinction event). This behaviour is common to all frameworks that include stochastic cell death (Roch, 2015) and where the extinction probability grows with step_size (Supplementary Fig. S2). We avoid the problem in practice by setting a sufficiently small step_size and a correspondingly large min_pop. In summary, at this stage, our system comprises a single clone, which is roughly constant in size and shows no clonal dynamics.

**Fig. 2.**
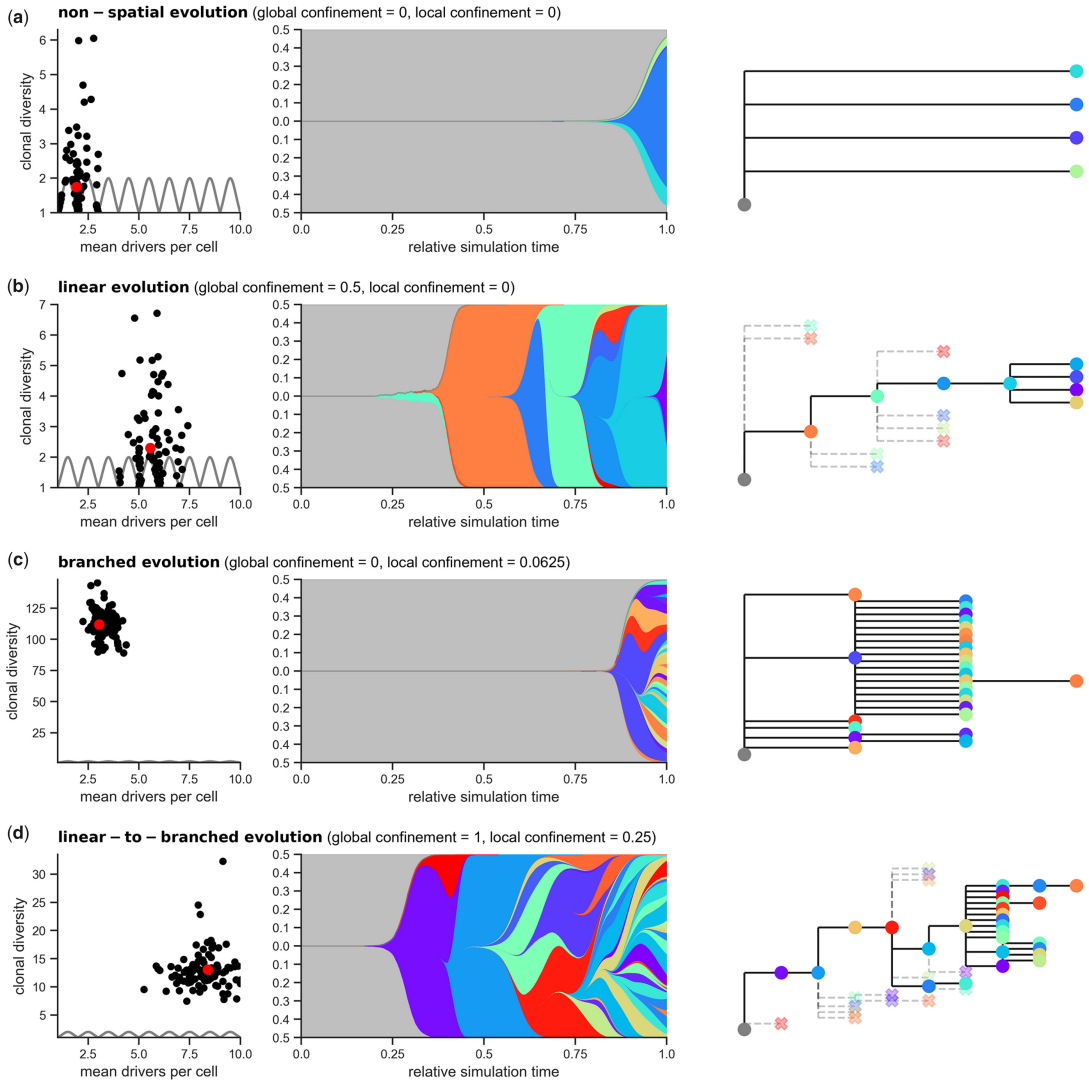
Combinations of global and local confinement can create different modes of evolution: (**a**) non-spatial evolution in the absence of confinement, (**b**) linear evolution after addition of global confinement, (**c**) branched evolution if only local confinement is enabled and (**d**) linear-to-branched evolution for the combination of local and global confinement. Left columns show the distribution of the mean number of drivers per cell and the clonal diversity for all 100 simulations per parametrization. The theoretical limit for clonal sweeps is shown as a grey line. The middle and the right columns show the clonal evolution of a representative simulation run, marked by a red circle, through a fish plot and a phylogenetic tree. For the phylogenetic trees, extinct clones (fewer than 1.000 alive cells) are crossed out. Note that for both the fish plots and phylogenetic trees, we selected only clones with a population fraction higher than 1%

### 2.3 Mutations

We next introduce mutations into the model. Because each mutation is unique, a new mutation always creates a new clone. We extend Equation (3) to


(4) $$B_{m}(c^{t})∼\text{Bin}(B_{b}(c^{t}),\theta_{\text{mut}}),\;$$

(5) $$mut(c^{t})=\min(B_{b}(c^{t}),B_{m}(c^{t})),\;$$

(6) $$c_{a}^{t+1}=c_{a}^{t}+B_{b}(c^{t})-B_{d}(c^{t})-mut(c^{t}).$$

For simplicity, we only allow one driver mutation per cell division. Since $\theta_{\text{mut}}\ll 1$, this does not affect the simulation in practice.

Mutated cells are removed from the group of new cells (Equation 6) and each of them spawns a new clone each with exactly one alive cell, such that for each $c^{t}\in C^{t}$: where *m^i^* is a new, unique mutation. The new clones are then added to the updated population, that is, $C^{t+1}=\cup \{\{c^{t+1}\}\cup \mathrm{children}(c^{t})|c^{t}\in C^{t}\}$.


(7) $$\mathrm{children}(c^{t})=\{(1,0,0,c_{M}\cup \{m^{i}\})|i\in \{1,\ldots ,mut(c^{t})\}\},$$

Our homeostatic system now consists of several clones of equal fitness. In such a system, the appearance and disappearance of clones is simply due to stochastic fluctuations and the constant accumulation of new neutral mutations (Supplementary Figs S2 and S3).

### 2.4 Fitness

We now leave the homeostatic scenario by introducing fitness-increasing driver mutations. The fitness increase *F*(*m*) that a new mutation *m* provides is drawn from a fitness distribution, dist∈{uni, exp, norm, const}, referring to the uniform, exponential, truncated normal and constant distributions, respectively. We use the term constant distribution as a shorthand for a single-element discrete uniform distribution. All the fitness distributions are set to a mean around $\theta_{\text{fit}}$ s.t.,


(8) $$F(m)∼\{\begin{array}{cc} U\{\theta_{\text{fit}},\theta_{\text{fit}}\}, & \;\text{if dist}=\text{const},\\ U_{\left[0,2\theta_{\text{fit}}\right]}, & \;\text{if dist}=\text{uni},\\ \;\text{Exp}(\theta_{\text{fit}}^{-1}), & \;\text{if dist}=\text{exp}\;,\\ N_{\left[0,\infty \right]}(\theta_{\text{fit}},\frac{\theta_{\text{fit}}}{2}), & \;\text{if dist}=\text{norm}. \end{array}$$

Here, $N_{\left[0,\infty \right]}$ represents the truncated normal distribution with a lower bound at 0 for which we chose a standard deviation of $\frac{\theta_{\text{fit}}}{2}$, in line with Bozic *et al.* (2010). Note that for the truncated normal distribution, a lower bound of 0 and $\sigma =\frac{\mu }{2}$ leads to an expected value of $1.027\cdot \theta_{\text{fit}}$. The exact shapes of the individual distributions are shown in Supplementary Figure S4.

To obtain the final fitness of the clone, we sum the effects of the individual mutations (in line with Fu *et al.*, 2022), using the function:


(9) $$fit(c^{t})=1+\sum_{m\in c_{M}^{t}}F(m).$$

In addition to the above, our model also supports multiple variants of the fitness accumulation, detailed in Supplementary Figure S5.

### 2.5 Global confinement

We now introduce quasi-spatial constraints into our model of a well-mixed population of cells. Driver mutations inevitably lead to exponential growth in the absence of limiting factors, which might include blood and nutrient supply or spatial constraints. We represent these constraints in an abstract manner using the *confinement* parameter $\theta_{\text{conf}}$. This global confinement acts in two ways. First, it limits the number of cells that can divide based on the size of the tumour. Second, it prevents some cells from disappearing after cell death, instead turning them into necrotic cells which continue to contribute to the overall size of the tumour.

To formulate global confinement, we create a geometrical representation of the tumour as a sphere (Fig. 1a and Supplementary Fig. S6). We fix the spatial scale of our model such that each individual cell has unit volume and the volume of the whole tumour at the time *t* is equal to the number of its alive and necrotic cells $C_{a}^{t}+C_{n}^{t}$. The tumour is thus divided into two regions, a proliferating *shell* and a quiescent *core*. Cells in the shell can divide and get removed when they die. Conversely, cells in the core cannot divide due to lack of space or resources, and turn into necrotic cells upon death. We denote $\text{shell-V}(C^{t})$ the volume of the shell and $\text{core-r}(C^{t})$ the radius of the core for the population contained in *C^t^*. Under the assumption of a perfect sphere, we can compute the fraction $\mathrm{frac}(C^{t})$ of the tumour volume occupied by the shell, relative to the shell volume $\text{shell-V}(C^{t})$ and the radius of the core $\text{core-r}(C^{t})$ as follows:


(10) $$\mathrm{frac}(C^{t})=\left\{ \begin{array}{cc} 0, & \text{if}\;C_{a}^{t}=0,\\ 1, & \text{else},\;\text{if}\;\theta_{\text{conf}}=0,\\ \max\left(\frac{\text{shell-V}(C^{t})}{C_{a}^{t}},1\right), & \text{else},\;\text{where:} \end{array}\right.$$

(11) $$\text{shell-V}(C^{t})=C_{a}^{t}+C_{n}^{t}-\frac{4}{3}\pi {\left(\text{core}\text{-r(}(C^{t})\right)}^{3}$$

(12) $$\text{core-r}(C^{t})=\max\left({\left(\frac{3}{4}\frac{C_{a}^{t}+C_{n}^{t}}{\pi }\right)}^{\frac{1}{3}}-\theta_{\text{conf}}^{-1},0\right).$$

Note that the width of the shell is given by $\theta_{\text{conf}}^{-1}$, meaning that lower confinement values lead to a larger shell and subsequently a larger proportion of proliferating cells (Supplementary Fig. S6b). In particular, for $\theta_{\text{conf}}\to 1$, the volume of the shell of a sphere approximates the surface area of that sphere, while for $\theta_{\text{conf}}\to 0$ the whole sphere is considered the shell, irrespective of its size. We then obtain the confined model:


(13) $$p_{\text{birth}}(c^{t})=\min\left(1,fit(c_{M}^{t})\cdot \mathrm{frac}(C^{t})\cdot \text{step}\_\text{size}\right),$$

(14) $$p_{\text{necro}}(c^{t})=1-\mathrm{frac}(C^{t}),$$

(15) $$B_{n}(c^{t})∼\text{Bin}\left(B_{d}(c^{t}),p_{\text{necro}}(c^{t})\right),$$

(16) $$c_{n}^{t+1}=c_{n}^{t}+B_{n}(c^{t}).$$

The shell fraction $\mathrm{frac}(C^{t})$ (Equation 10) limits the number of new cells (Equation 13) by limiting the birth probability. The death probability is not affected by the shell fraction; however, it does separate necrotic and removed cells. Note that the total number of necrotic cells is conditional on step_size through $B_{d}(c^{t})$ as given in Equation (1).

### 2.6 Local confinement

Global confinement as defined above represents a competition of the whole cell population for shared resources. Additionally, in some tumours, local space or resource restrictions might apply, as for example in the case of breast cancer arising from the mammary glands, where smaller localized tumour cell populations press against the glandular epithelium (Lomakin *et al.*, 2022).

We represent these local spatial constraints in our framework by adding a second (local) confinement term for each clone individually. When applied without global confinement, it limits each clone by its own population size (Fig. 1b). When combined, the global confinement is modulated by the size of the clone (Fig. 1c).

To compute this effect, we introduce an additional function *local* that is equal to the function *frac* (Equation 10) where the parameter $\theta_{\text{local}}$ is used in place of $\theta_{\text{conf}}$, which further limits the birth probability of each clone $c^{t}\in C^{t}$ such that: where [x/y]f denotes substituting the parameter *x* in function **f** for *y*.


(17) $$\mathrm{local}=[\theta_{\text{conf}}/\theta_{\text{local}}]\mathrm{frac},$$

(18) $$p_{\text{birth}}(c^{t})=\min\left(1,fit(c^{t})\cdot \mathrm{frac}(C^{t})\cdot \mathrm{local}(c^{t})\cdot \text{step}\_\text{size}\right),$$

(19) $$p_{\text{necro}}(c^{t})=1-\mathrm{frac}(C^{t})\cdot \mathrm{local}(c^{t}),$$

Observe that if we set $\theta_{\text{local}}=0$, the model is equal to the one in Section 2.5, with $\theta_{\text{conf}}=0$ to Section 2.4, with $\theta_{\text{fit}}=0$ to Section 2.3 and with $\theta_{\text{mut}}=0$ to Section 2.2.

### 2.7 Model configuration

We aim to simulate realistically sized tumours with $∼10^{9}$ cells, corresponding to a tumour of $>1\;{\text{cm}}^{3}$ in size (Del Monte, 2009). We set the minimum population size min_pop for a simulation to be considered to 1000 cells. max_pop is then derived from min_pop as $1000\cdot 2^{20}∼10^{9}$, hence population doubling occurs 20 times starting from min_pop. To calibrate cell turnover step_size, we used the homeostatic model (Section 2.2) for a starting population size of 100 cells and evaluated step_size∈{0.1,0.05,0.01,0.005}. We found that for step_size=0.01 there was no extinction event within max_steps=1000 for any of the 1000 replicates considered, and we thus selected 0.01 as a turnover value for all further simulations. We fixed the limiting variable for the maximum number of generations max_steps to 10^6^, such that without a mutation the initial cell divides on average $10^{6}\cdot 0.01=10^{4}$ times. Assuming a typical division cycle of 24 h, this parametrization would correspond to ∼27 years of real time. In all subsequent simulations, the execution reached the maximum population size before reaching the maximum number of generations.

As we are mainly interested in the effect of confinement, unless stated otherwise we fix the mutation probability and mean fitness gain of a single driver at $\theta_{\text{mut}}=2\cdot 10^{-5}$ and $\theta_{\text{fit}}=0.1$ in line with the literature (Noble *et al.*, 2022; West *et al.*, 2021). We additionally set dist=exp  as in Noble *et al.* (2022).

For the robustness analysis in Sections 3.1and 3.2, we later perturbed these two parameters, testing $\theta_{\text{fit}}\in \{0.01,0.05,0.1,0.15,0.2\}$ and $\theta_{\text{mut}}\in \{2\cdot 10^{-6},\;10^{-5},\;2\cdot 10^{-5},\;10^{-4},\;2\cdot 10^{-4}\}$.

### 2.8 Population metrics

To evaluate the individual simulation runs and to compare their clonal behaviour to prior work as well as experimental data, we use two previously introduced metrics: the *mean number of drivers per cell* and the *clonal diversity index* (Noble *et al.*, 2022). To speed up computation of summary statistics, we only consider clones larger than a minimum fraction cutoff of the population (${\tilde{C}}^{t}=\{c^{t}\in C^{t}\;|\;c_{a}^{t}\geq C_{a}^{t}\;\cdot \;\text{cutoff}\}$) and only calculate the metrics at every time step at which the population of alive cells doubles. The Fish plots (e.g. Fig. 2a–d) are not affected by the cutoff, but only display clones that reached at least 1% of the population at some point.

The *mean number of drivers per cell* $\bar{d}$ tracks the mutational burden of the growing tumour and is defined as


(20) $$\bar{d}({\tilde{C}}^{t})=\sum_{c^{t}\in {\tilde{C}}^{t}}\left(|c_{M}^{t}|\cdot c_{a}^{t}\right)\cdot {\left({\tilde{C}}_{a}^{t}\right)}^{-1}.$$

The *clonal diversity index D* reflects the total number of equally sized clones and their size and is based on the inverse Simpson index defined as expected:


(21) $$D({\tilde{C}}^{t})={\left(\sum_{c^{t}\in {\tilde{C}}^{t}}{\left(c_{a}^{t}\cdot {\left({\tilde{C}}_{a}^{t}\right)}^{-1}\right)}^{2}\right)}^{-1}.$$

This measure has been shown to have a lower boundary of 1 and to be robust to the presence or absence of small populations (Noble *et al.*, 2022).

### 2.9 Comparing with real tumour data

To compare our simulations to real data, we used six datasets from the following cancer types (Noble *et al.*, 2022): acute myeloid leukaemia (AML, single-cell DNA sequencing) (Morita *et al.*, 2020), kidney (clear cell renal cell carcinoma, multi-region whole-exome sequencing) (Turajlic *et al.*, 2018), mesothelioma (multi-region whole-exome sequencing) (Zhang *et al.*, 2021), breast (triple-negative breast cancer, single-cell RNA sequencing) (Minussi *et al.*, 2021), lung (non-small cell lung cancer, multi-region whole-exome sequencing) (Jamal-Hanjani *et al.*, 2017), and uveal (uveal melanoma, single-cell RNA sequencing) (Durante *et al.*, 2020). One additional dataset of whole-genome sequenced breast cancer was discarded since it only had three samples available.

To fit our simulations to the data, we considered all combinations for $\theta_{\text{conf}}$ and $\theta_{\text{local}}$ in the range of [0,0.125,0.25,0.5,1,2] each. For every combination, we simulated 100 runs and compared the simulation with the data using a score based on the Iterative Closest Point algorithm. To this end, we calculated the shortest Euclidean distance between the simulated data and the real data in the two-dimensional space defined by the mean number of drivers per cell and the clonal diversity. The final score (S) is then defined as the mean shortest distance for each simulated point, plus the mean shortest distance for each data point, i.e. where *X_s_* are the clones in a simulation and *X_d_* in a real dataset.


(22) $$S(X_{s},X_{d})=\frac{1}{\left|X_{s}\right|}\sum_{x_{s}\in X_{s}}E(x_{s},X_{d})+\frac{1}{\left|X_{d}\right|}\sum_{x_{d}\in X_{d}}E(x_{d},X_{s}),$$

(23) $$E(x,Y)=\min{\left\{{\left(\bar{d}(x)-\bar{d}(y)\right)}^{2}+{\left(D(x)-D(y)\right)}^{2}\right)}^{\frac{1}{2}}|y\in Y\},$$

Additionally, we also analysed the fit of the spatial simulation created by Noble *et al.* (2022) to the six datasets. To this end, we downloaded all simulation results from https://github.com/robjohnnoble/ModesOfEvolution, which included model parameters as well as the clonal diversity and mean number of drivers per cell for every simulation run. We then calculated a score (S) for every simulation parametrization and cancer type using Equation (22). To allow for fair comparisons, we choose the parametrization with the best overlap for every cancer type by varying all model parameters available in the data: spatial constraint, mutation probability, fitness gain per driver and deme size.

### 2.10 Smith implementation and performance

SMITH has been implemented in C# as an open-source package under the MIT license and is available at https://bitbucket.org/schwarzlab/smith with pre-compiled binaries for Windows, Linux and MacOS. Version 1.1 is used in this article and the code and data to reproduce the figures can also be found at https://doi.org/10.5281/zenodo.6885040. The Fish (Muller) plots were generated using the accompanying open-source Python library *PyFish*, available at https://bitbucket.org/schwarzlab/pyfish.

SMITH performance was evaluated against *tumopp*, a high-performance simulator of on-lattice cancer growth (Iwasaki and Innan, 2017). Both SMITH and *tumopp* were parameterized with the same driver mutation rates and effects, and performance was compared on population sizes ranging from 10^4^ to 10^9^ cells with 10-fold increases. Global confinement was set to 1 to mimic the surface-growth model of *tumopp*. Overall, the SMITH simulation proved to be about 1000-fold faster than *tumopp*. The maximum population *tumopp* was able to simulate with 32 GB of memory was 10^7^ cells. Simulation of a tumour population of 10^7^ cells took 2341.55 s in *tumopp* compared with 1.32 s in SMITH with confinement and 0.14 s without confinement. For a 1 billion model, the average runtime was 8.95 s without and 256.47 s with global confinement, respectively (Supplementary Fig. S7). The memory usage was less than 1 GB.

## 3 Results

### 3.1 Global and local confinement recreates common modes of cancer evolution

Recent investigations into common patterns of cancer evolution have revealed several major ‘modes’ of evolution. While non-spatial tumours, including lymphomas and leukaemia, are characterized by few driver events and rapid clonal expansions (Ferrando and López-Otín, 2017), in spatially organized solid tumours more diverse evolutionary patterns have been described, including ‘neutral’, ‘linear’ and ‘branched’ evolution (Vendramin *et al.*, 2021). We hypothesized that these evolutionary modes are driven by varying local and global constraints acting on these tumours, including available physical space, nutrient availability and blood supply. To test this hypothesis within the scope of our model, we fixed the mutation probability and fitness sampling and accumulation to literature derived default values (Section 2.7) and only varied local and global confinement (Supplementary Figs S8 and S9).

In the absence of confinement, our model is mathematically equivalent to a traditional, non-spatial branching process (Section 2.1). By setting the average fitness increase per mutation to zero, we obtain a fully neutral growth pattern (Supplementary Fig. S3). This mode is characterized by variants that can reach fixation purely through genetic drift but rarely observed in real-life tumours (Sottoriva *et al.*, 2015).

**Fig. 3.**
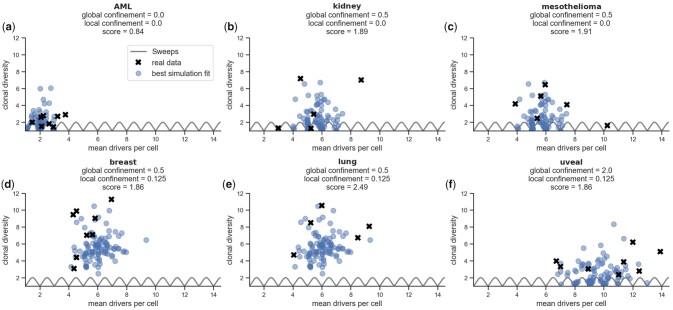
Different values for global and local confinement recreate the clonal evolution of different tumour types. For every dataset, we show the mean number of drivers per cell and the clonal diversity both for the real tumour samples (black crosses) and the 100 simulations with the global and local confinement combination that best fit the dataset (blue circles)

With the default fitness increase of 0.1 per driver mutation and in the absence of confinement, SMITH simulations are characterized by a low number of clones that grow exponentially (Fig. 2a), matching the unconstrained evolution of non-spatial tumours, in particular leukaemia (Lee and Bozic, 2022). These simulations are also characterized by low clonal diversity (median of 1.3) and a low mean number of drivers (median of 2.0) as earlier clones dominate the makeup of the tumour. Without spatial restrictions, these simulations reach the final size of 1 billion cells faster than any parametrization considered, in line with observations of exponential growth in leukaemia (Lee and Bozic, 2022).

Intriguingly, adding global confinement to the model triggers development of the typical ‘linear’ or ‘ladder-like’ evolutionary patterns (Schwarz *et al.*, 2015; Vendramin *et al.*, 2021) characterized by repeated clonal sweeps where individual subclones successively take over the cell population (Fig. 2b). Global confinement here simulates a global competition for space and resources which slows the growth of existing clones and favours well-adapted clones with higher fitness, creating a tumour with a high number of driver events. Consequently, we observe consistent accumulation of driver mutations (median of 5.7) at the expense of diversity (median of 1.9).

In contrast, adding only local confinement to the model leads to rapid and parallel clonal expansions and a corresponding high clonal diversity (median of 112, Fig. 2c). Local confinement thereby constricts the growth of each clone individually, as might, for example, be caused by local tissue structures such as glands (Section 2.6). Consequently, many subbranches co-exist in the phylogeny of these tumours and clones expand simultaneously creating a very diverse tumour with a highly ‘branched’ phylogeny. Arguably, local confinement alone is unlikely to exist in real-world tumours, as it omits the global limiting factor of shared resources.

More realistically, real-world tumours will likely be constrained by both global and local growth restrictions and show patterns somewhere in between the linear and branched evolutionary modes. Notably, recent research has identified widespread transitions in tumours wherein mutations first occur successively in the early stage of tumour evolution. This is followed by branching of the evolutionary trees in the latter stage (Baslan *et al.*, 2022; Lomakin *et al.*, 2022). In our model, when global confinement is larger than local confinement ($\theta_{\text{conf}}>\theta_{\text{local}}>0$), we also find a mixture of the linear and branched modes which we refer to as ‘linear-to-branched evolution’ (Fig. 2d). These simulations evolve in two distinct phases. In the beginning, clones are small and global confinement typically leads to several early clonal sweeps. As the population grows, local confinement begins to exhibit a greater influence, disproportionately slowing the growth of larger clones and causing smaller clones to appear—a fully branched tumour emerges. The linear-to-branched mode of evolution shows both signs of rapid emergence of new clones (median of 8.2 mean number of drivers) and high clonal diversity (median of 12.6) reminiscent of real, spatially organized tumours (see below).

The observed results were robust with regard to changes to the mutation probability (Supplementary Fig. S10) and the fitness advantage gained by a single driver (Supplementary Fig. S11). This robustness was to be expected as it was also observed in other simulation frameworks (Fu *et al.*, 2022; Noble *et al.*, 2022; West *et al.*, 2021).

In summary, varying local and global confinement allows us to recreate the evolutionary patterns observed in non-spatial and spatially organized tumours. Global and local confinement thereby directly affects the mean number of drivers and the extent of clonal diversity (for detailed analysis, see Supplementary Fig. S8).

### 3.2 Confinement models recapitulate the clonal evolution of real-life tumours

Next, we evaluated the ability of our model to fit real data from six different cancer types (AML, lung, kidney, uveal, mesothelioma and breast; Supplementary Fig. S12) derived by Noble *et al.* (2022) (Section 2.9). To this end, we varied the global and local confinement, each in the range of [0,0.125,0.25,0.5,1,2], with the model setup as given in Section 2.7. For each parameter combination, we created 100 simulations and found the optimal confinement values per dataset by minimizing the score function (Equation 22), which takes into account the mean number of drivers per cell and the clonal diversity (Section 2.9). For visualization of the optimal confinement values, see Supplementary Figure S13.

In AML, as expected, the optimal fit was achieved by setting both global and local confinement to zero, corresponding to a traditional non-spatial simulation (Fig. 3a), appropriate for non-spatially organized cancers (Lee and Bozic, 2022).

Most other spatially organized tumour types (kidney, mesothelioma, breast and lung) are characterized by a moderate number of drivers per cell (median of 4.9 for breast, 5.3 for kidney, 5.8 for mesothelioma and 6.0 for lung) and hence were fit best by a global confinement value of 0.5 (Fig. 3b–e).

Additionally, tumours of the kidney and mesothelioma datasets had a comparatively low median clonal diversity (2.9 and 4.1, respectively) resulting in a best fit in the absence of local confinement (Fig. 3b, c) and leading to ‘linear’ evolutionary patterns marked by repeated clonal sweeps (see Fig. 2c). In fact, two out of the five samples in the kidney dataset and one sample in the mesothelioma dataset are currently mid-sweep as demonstrated by a clonal diversity of ∼1.

In contrast, the samples from the lung and breast cohorts were best fit with local confinement of $\theta_{\text{local}}=0.125$ (Fig. 3d, e) to account for their relatively high median clonal diversity of 8.1 and 8.0, respectively. The comparatively high local confinement in these tumours might correspond to localized growth into separate mammary glands for breast cancer and alveoli for lung cancer. With the combination of global and local confinement, these samples are best described with the linear-to-branched clonal evolution produced within our model (Fig. 2d).

Finally, the samples in the uveal cancer dataset have a comparatively high number of drivers (median of 11.2) which is recapitulated in our model through the highest global confinement value of 2, corresponding to half of the cells in the outer layer dividing, in combination with local confinement of 0.125 (Fig. 3f). Interestingly, uveal melanomas indeed show unique physiological characteristics in that they are slowly growing and heavily restricted to the eye (Kaliki and Shields, 2017).

Furthermore, we evaluated the robustness of our estimates and found that the scores for each dataset were robust to variations in fitness mean and mutation probability (Supplementary Fig. S14) as described in Section 3.1 (Supplementary Figs S10 and S11).

Next, we compared our best fits to the ones obtained from the explicitly spatial model of (Noble *et al.*, 2022) using the same score function (Equation 22) as above (Section 2.9). As expected, for the AML data, SMITH and the *Noble* model provided similarly good fits (Supplementary Fig. S15), owing to the fact that in the absence of confinement and spatial organization both models describe a non-spatial branching process. For the spatially organized cancer types, our model outperforms the Noble *et al.* model for all cancer types (kidney, lung, uveal and mesothelioma) with the exception of breast cancer, for which both models generate a similarly good fit (with 1.67 and 1.86, respectively, Supplementary Fig. S15).

Interestingly, in the kidney cancer dataset, two out of the five samples are currently mid-clonal sweep, which is marked by a clonal diversity of ≤1.5. Due to the spatial arrangement of the Noble *et al.* simulation, clonal sweeps are extremely rare to occur (less than 2% of the 2.700 simulations of the invasive glandular mode have clonal diversity ≤1.5), whereas they frequently occur in our simulation framework (39% for $\theta_{\text{conf}}=0.5,\theta_{\text{local}}=0$).

### 3.3 Population size affects the evolutionary dynamics of tumours

Due to computational limitations, current explicit spatial models do not exceed 10^7^ individual simulation voxels: 10^6^ for Noble *et al.* (2022) and $8\cdot 10^{6}$ for Fu *et al.* (2022), West *et al.* (2021) and Sottoriva *et al.* (2015). In some of these models, larger population sizes can be reached only by making each voxel represent a clone of a fixed size (e.g. 10^4^ cells per gland in Sottoriva *et al.*). By replacing explicit spatial representations with the confinement mechanics, we can simulate populations beyond 1 billion cells, corresponding to tumour sizes commonly encountered in the clinic at initial diagnosis (Del Monte, 2009; Welch *et al.*, 2016). Leveraging the efficiency of SMITH, we investigated the advantages of simulating large populations and assessed how ITH changes over time in the course of repeated simulations.

We observed that for all scenarios that include global or local confinement the mean number of drivers grows logarithmically with the population size (Supplementary Fig. S16), demonstrating that newly appearing clones eventually overtake the population. This logarithmic increase is of particular interest for the uveal cancer dataset which contains up to 14 mean drivers per cell, which would be difficult to reach in populations as small as 10^6^ cells.

In the absence of confinement (i.e. non-spatial), tumours develop mostly one clearly dominant clone with only a few competitors. Clonal diversity therefore barely reaches a score of 2 for the final size of 10^9^ cells. If only global confinement is applied, clonal diversity plateaus after an initial surge at around 10^6^ cells, after which clonal sweeps increase in frequency and repeatedly reduce the clonal diversity to 1. Conversely, with only local confinement active, clonal diversity continuously rises until the end of the simulation, since the growth rate of larger clones is dampened relative to smaller clones. When both local and global confinements are employed, we first see clonal sweeps until about 10^7^ cells (corresponding to ∼100 cells in diameter for a solid tumour), after which we increasingly observe a branching behaviour and diversification of the tumour. Interestingly, this transition from a ‘linear’ progression to a branching in the phylogeny would be missed in simulations smaller than 10^7^ cells.

Furthermore, when investigating individual trajectories in the presence of global confinement, we observe extensive heterogeneity in their behaviour, governed by alternating periods of clonal sweeps and diversification (Supplementary Fig. S17).

## 4 Discussion

The effects of spatial restrictions have long been observed in explicit spatial models, in particular in West *et al.* (2021), where the authors investigated both local and global growth limitations. Additionally, other authors postulated that it is indeed the explicit spatial representation from which ITH emerges (Fu *et al.*, 2022; Noble *et al.*, 2022).

Here, we have presented SMITH, a new simulation model for cancer evolution based on confinement, which limits population growth globally and locally in a non-linear way. By combining different values for global and local confinement, SMITH is able to reproduce commonly observed modes of cancer evolution including non-spatial, neutral, linear, branched as well as linear-to-branched evolution. Adjusting global and local confinement thereby directly affects the mean number of drivers per cell and the clonal diversity of a tumour, respectively. Additionally, we have demonstrated that specific combinations of global and local confinement can recreate the behaviour of real-life tumours. However, the available data are limited in size and additional data are needed to ascertain whether this observation generalizes to other cancer entities.

Other extensions to branching processes have been proposed, including a logistic-growth model which focuses on modelling specific hallmarks of cancer (Nagornov and Kato, 2020). Others provided spherical interpretations of tumours in mathematical models of tumour growth (Dassios *et al.*, 2012; Paterson *et al.*, 2016). In implementing our confinement mechanism, we worked under the assumption of a spherical tumour, which gives rise to the $\frac{2}{3}$ power law (Section 2.5). While this might not be suitable for all kinds of tumours, it should be noted that more general formulations of the confinement mechanism could easily be derived, as long as the growth rate scales non-linearly with the population size (see also Talkington and Durrett, 2015).

One decisive advantage of our model is its speed advantage over explicit spatial models, which are commonly run on high-performance computers in parallel with cell populations limited to $∼10^{7}$ (Rosenbauer *et al.*, 2020). In contrast, SMITH performs a single simulation up to 10^9^ cells in minutes on a desktop computer, which allows for the analysis of the variability of evolutionary trajectories under a variety of different parametrizations.

Besides confinement, fitness mean and mutation rate, other modelling choices might influence the outcome of simulations, most prominently the choice of the fitness distribution. Other authors have proposed the use of a constant (West *et al.*, 2021), uniform (Fu *et al.*, 2022), normal (Sottoriva *et al.*, 2015) and exponential (Noble *et al.*, 2022) distribution from which to draw fitness values (Section 2.4). We systematically explored the effect of the choice of fitness distribution on the simulation results of SMITH (Supplementary Fig. S18). In the absence of confinement, corresponding to non-spatial evolution, the number of drivers and clonal diversity were largely unaffected, with the exception of the constant distribution (Supplementary Fig. S18) and in line with previous findings (McFarland *et al.*, 2013). In contrast, the choice of fitness distribution did influence the number of mean drivers per cell and the clonal diversity in the presence of spatial constraints but without fundamentally changing the effect of local and global confinement on the simulation outcomes. Further investigations into these and other commonly made modelling choices are warranted to improve comparability between models and parameters in the future. SMITH, with its fast computation, simplicity and the ability to capture different data types and evolutionary modes are well poised to serve as a testbed for such analyses in the future.

In this work, we have developed a minimal model of tumour evolution centred around the concepts of global and local spatial restrictions. By linking our model to established modes of tumour evolution as well as real-life tumours, we propose local and global confinement as a major determinant of ITH and the clonal evolution of tumours.

## Supplementary Material

btad102_Supplementary_Data
