# Supplementary material for: SMITH: spatially constrained stochastic model for simulation of intra-tumour heterogeneity
Source: Bioinformatics. 2023 Feb 24;39(3):btad102. doi: 10.1093/bioinformatics/btad102 (PMC10010604; doi:10.1093/bioinformatics/btad102)
Supplement: btad102_Supplementary_Data [file btad102_supplementary_data.pdf]

SMITH: Spatially Constrained Stochastic Model for Simulation of Intra-tumour Heterogeneity

Supplementary Material

Adam Streck[1,2], Tom L. Kaufmann[3,2,1], and Roland F. Schwarz[2,3,1]

[1] *Berlin Institute for Medical Systems Biology, Max Delbrück Center for Molecular Medicine in the Helmholtz Association, Berlin, Germany*

[2] *Institute for Computational Cancer Biology (ICCB), Center for Integrated Oncology (CIO), Cancer Research Center Cologne Essen (CCCE), Faculty of Medicine and University Hospital Cologne, University of Cologne, Cologne, Germany*

[3] *BIFOLD - Berlin Institute for the Foundations of Learning and Data, Berlin, Germany*

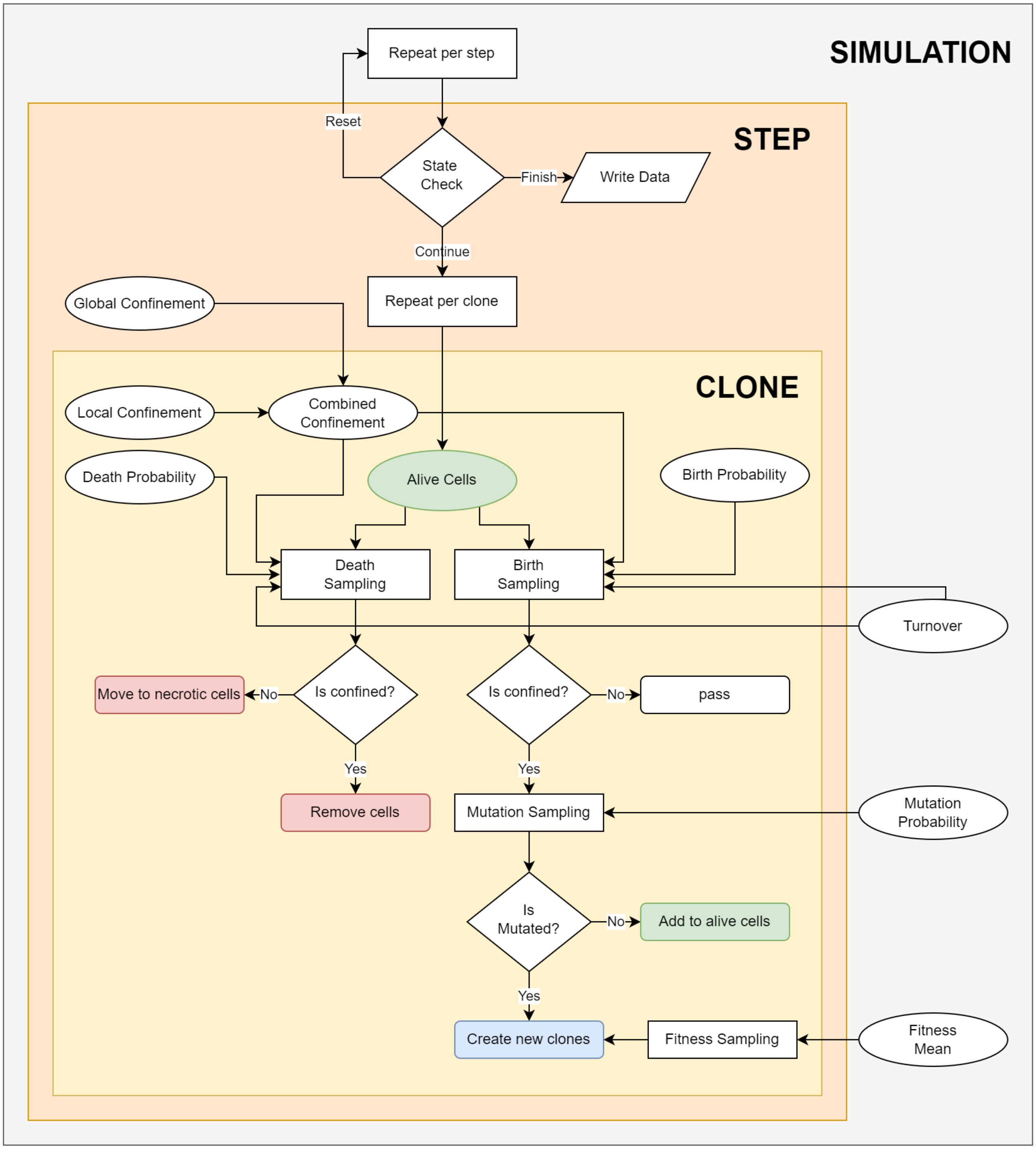

Supplementary Figure 1: Simulation flow. A single run is repeated in iterative steps, with each step evaluating each clone. The "Is confined" decision is made in accordance with Eq. 13, 14 of the main text.

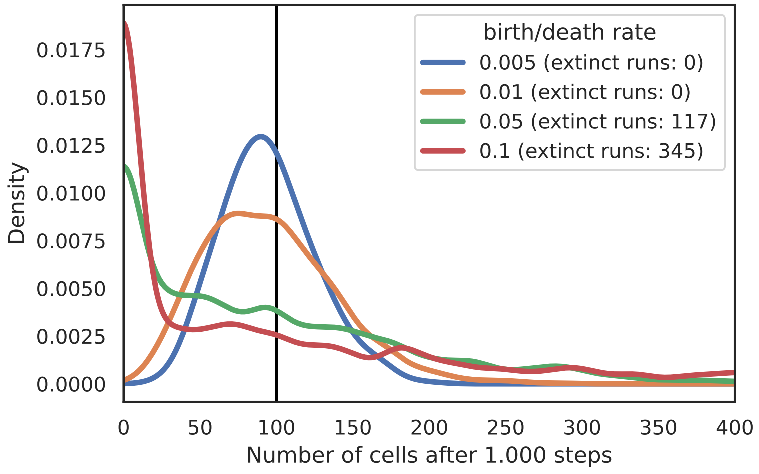

Supplementary Figure 2: Homeostasis without sations: We simulate 1000 runs each for four separate values of *step_size* each starting at 100 cells. For 0.05 and 0.1 the turnover is high enough that we observe extinction of the population in multiple runs.

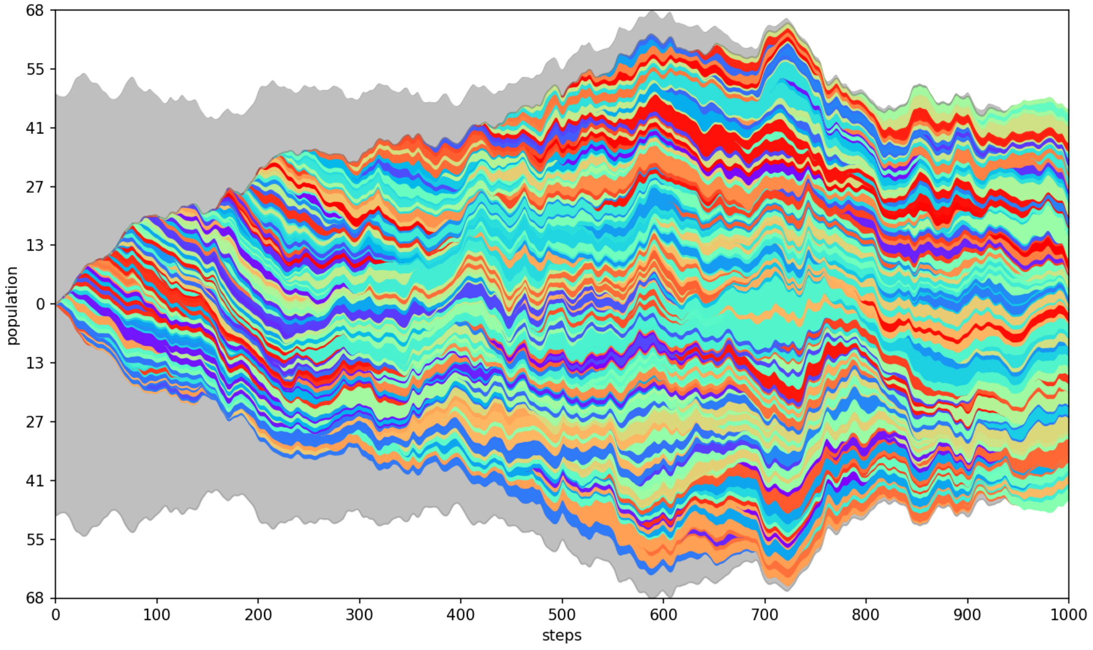

Supplementary Figure 3: An illustration of mutation accumulation over 1000 steps with homeostatic population of 100 cells ($C^0 = \{(100, 0, \{m\})\}$) and the mutation probability $\theta_{\text{mut}} = 1$. Each colour represents the size of a population of a single clone. We can see that the original population (grey) disappears as a result of mutation accumulation during turnover. Some of the newly created clones disappear as well while others branch into further clones.

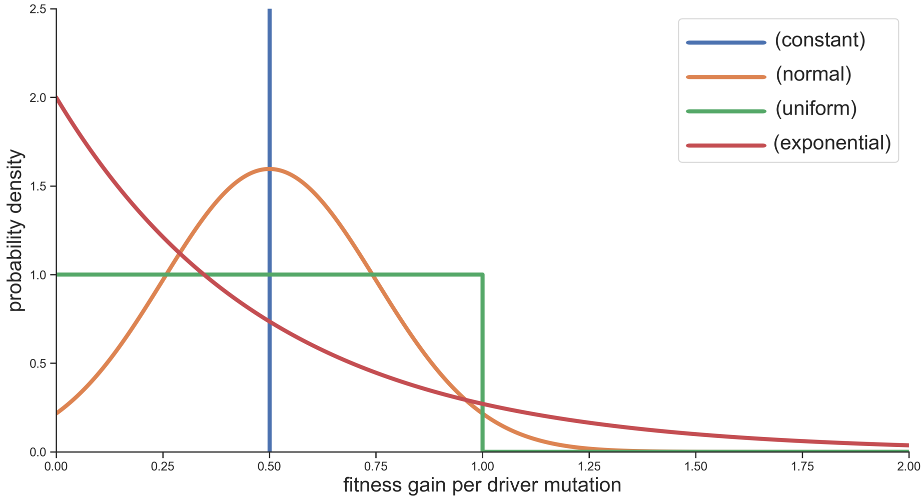

Supplementary Figure 4: Distribution of fitness around the mean $\theta_{\text{fit}} = 0.5$ for the random variables C^θ (constant), N^θ (normal), U^θ (uniform), and E^θ (exponential).

$$\mathbf{fit}(c^t) = \begin{cases} \prod_{m \in c^t_M} (1 + F(m)) & \text{, if } acc = mul, \\ \mathbf{limit}(c^t_M, |c^t_M|) & \text{, if } acc = asy, \\ 1 + \sum_{m \in c^t_M} F(m) & \text{, if } acc = add. \end{cases}$$

$$\mathbf{limit}(c_M, i) = \begin{cases} 1 & \text{, if } i \leq 0, \\ \mathbf{limit}(c_M, i-1) \left(1 + F(c_{M(i)})(1 - \frac{\mathbf{limit}(c_M, i-1)}{10})\right) & \text{, else.} \end{cases}$$

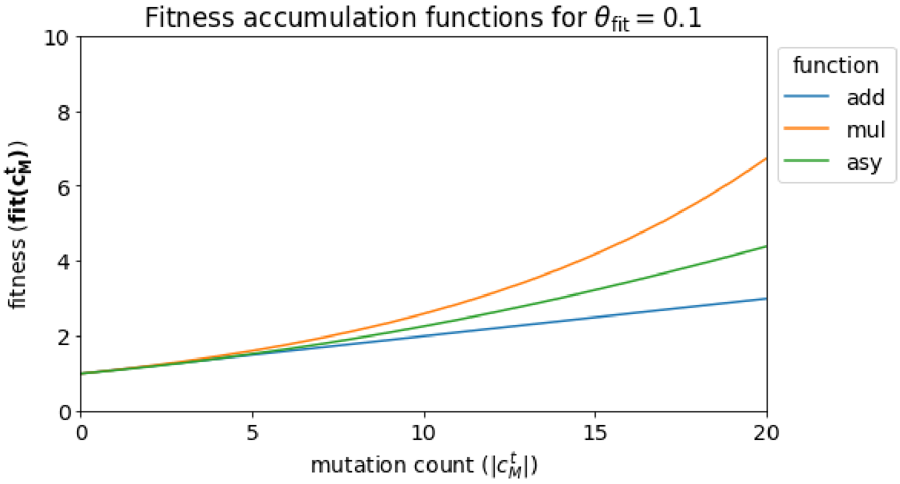

Supplementary Figure 5: Top: We have implemented three accumulation functions: multiplicative (*mul*), asymptotic (*asy*), and additive (*add*) Middle: The asymptotic function **limit** given in [Noble et al., 2022]. The function is calculated dependent on the order in which mutations have been accumulated. To this end we use the notation $c_{M(i)}$ to denote the i-th mutation in c_M. Bottom: Comparison of the accumulation functions. Notice that for the fitness selected in the text, the asymptotic function behaves in almost linear fashion.

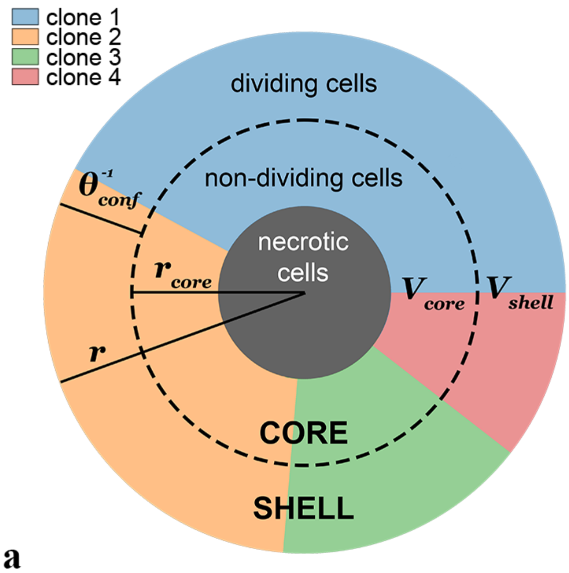

a

$$r = \left(\frac{3}{4}\frac{C_a + C_n}{\pi}\right)^{\frac{1}{3}}$$

$$r_{core} = r - \theta_{\mathrm{conf}}^{-1}$$

$$V_{core} = \frac{4}{3}\pi r_{core}^3$$

$$V_{shell} = C_a + C_n - V_{core}$$

b

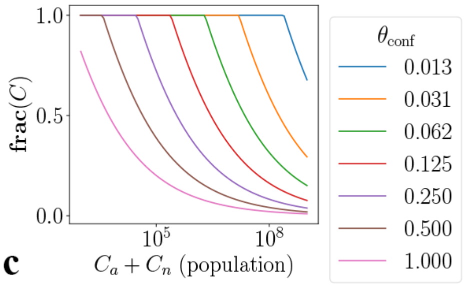

c

Supplementary Figure 6: A numerical illustration of the global confinement. a) corresponds to the Fig. 1 of the main text. b) shows how the individual values are calculated. c) illustrates the relationship between the population size, confinement and the fraction between the shell and the alive population.

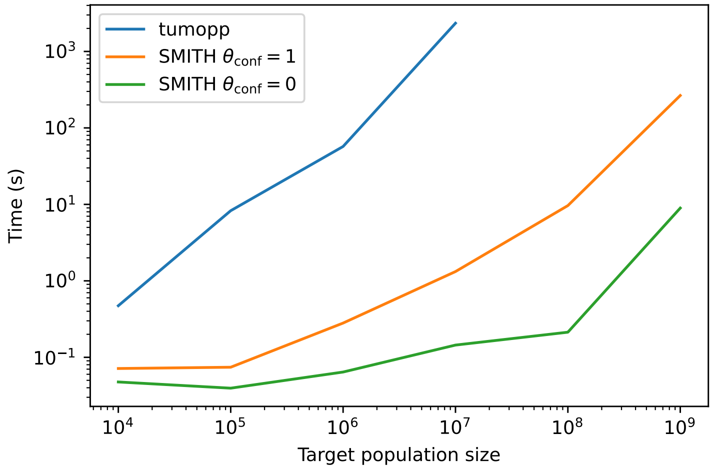

a

```
tumopp -N1000000 -b 1.1 -d 1.0 --ub 0.00001 --mb 0.1 --sb 0.05
```

b

```
{
  "Seed": -1,
  "StartMut": 1,
  "StartPop": 1,
  "Reps": 10,
  "MaxPop": 1000000000,
  "MaxSteps": 1000000,
  "MinPop": 1,
  "Turnover": 0.01,
  "MutationProb": 0.00002,
  "DriverProb": 1,
  "FitnessMean": 0.1,
  "ConfGlobal": 1,
  "ConfLocal": 0,
  "FitnessAcc": 1,
  "FitnessDist": 1,
  "FitnessEffect": 0,
  "CutOff": 0.0001
}
```

c

Supplementary Figure 7: **a)** Performance comparison between SMITH and tumopp for population sizes between $10^4 - 10^9$. For 10^8, tumopp exceeded 16GB of memory and did not finish. A model with 10^{-5} driver probability and birth-increasing effect sampled from a normal distribution with mean of 0.1 was selected. **b)** The execution command for tumopp. **c)** Execution configuration for SMITH. Confinement of 1 was selected to mimic the surface growth model of tumopp (orange), and 0 to demonstrate the performance on a non-spatial model (green).

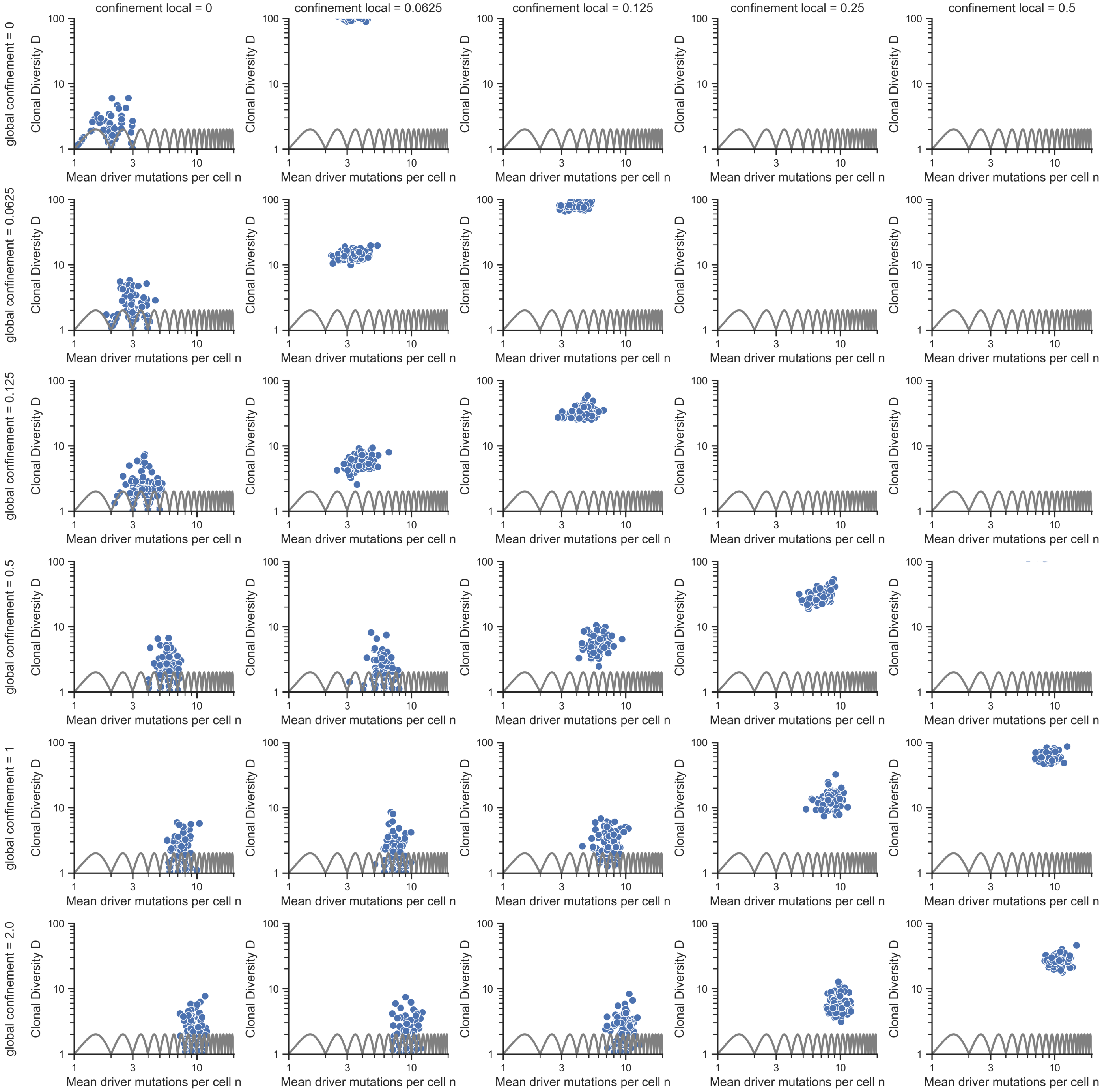

Supplementary Figure 8: Effect of the global and local confinement on the mean number of drivers per cell and the clonal diversity. For every combination of local and global confinement 100 simulations were run. Note that for some confinement values, the clonal diversity exceeded 100 and the simulations are not thus not shown. It's clearly visible how increasing the global and local confinement leads to an increased mean number of drivers and clonal diversity, respectively.

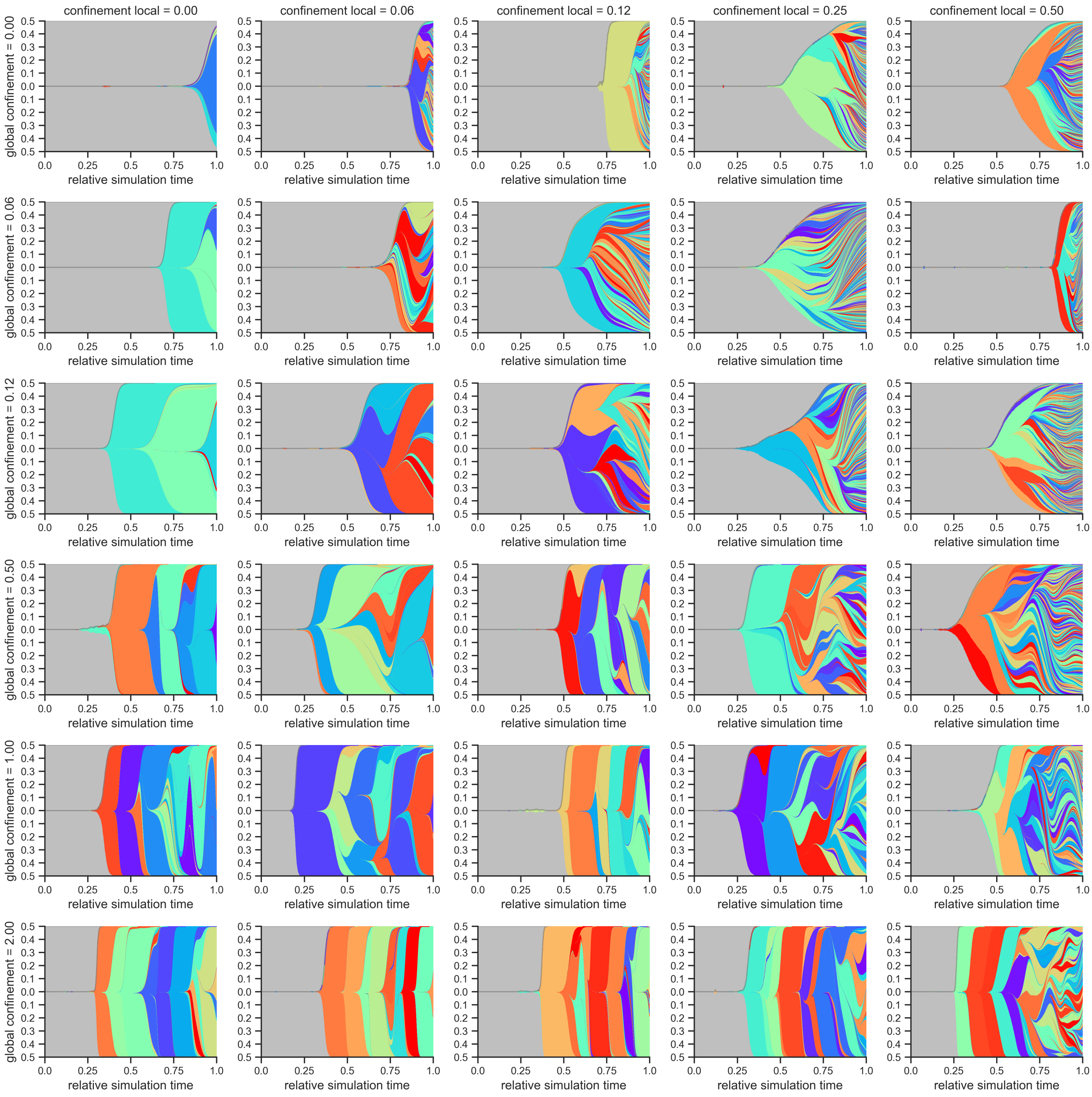

Supplementary Figure 9: Example fish plots for different combinations of local and global confinement. For every combination of local and global confinement, the most representative simulation run was selected based on the mean number of drivers and the clonal diversity (similar to Main Figure 2).

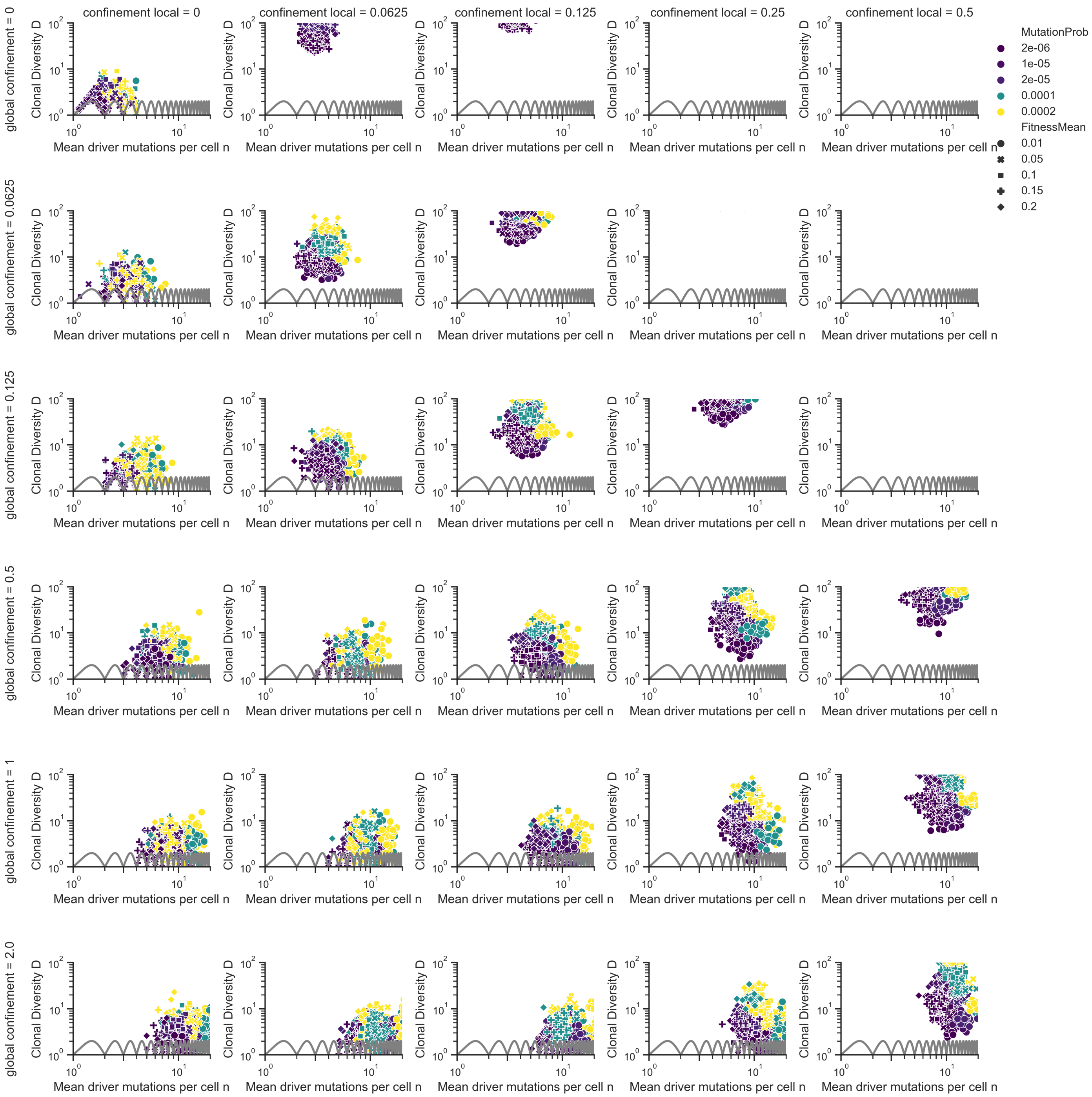

Supplementary Figure 10: Effect of the mutation probability on the mean number of drivers and the clonal diversity. In contrast to Supp. Fig. 8, the mutation probability and the fitness advantage gained by a single driver was varied in the ranges $\theta_{\text{fit}} \in \{0.01, 0.05, 0.1, 0.15, 0.2\}$ and $\theta_{\text{mut}} \in \{2 \cdot 10^{-6}, 10^{-5}, 2 \cdot 10^{-5}, 10^{-4}, 2 \cdot 10^{-4}\}$. With 100 simulations per parameter combination, every panel shows 2500 simulations.

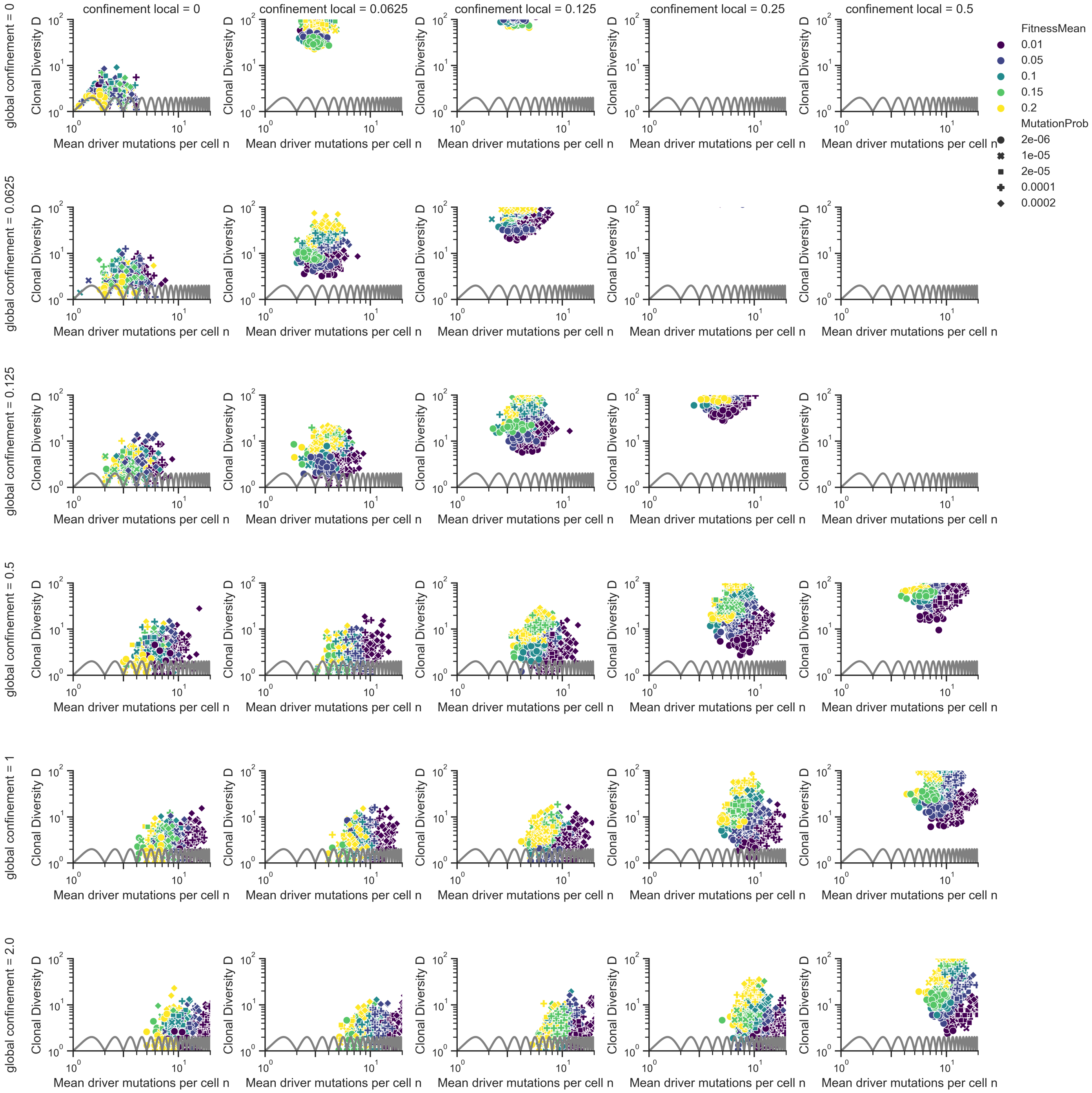

Supplementary Figure 11: Effect of the mean fitness gain per driver on the mean number of drivers and the clonal diversity. In contrast to Supp. Fig. 8, the mutation probability and the fitness advantage gained by a single driver was varied in the ranges $\theta_{\text{fit}} \in \{0.01, 0.05, 0.1, 0.15, 0.2\}$ and $\theta_{\text{mut}} \in \{2 \cdot 10^{-6}, 10^{-5}, 2 \cdot 10^{-5}, 10^{-4}, 2 \cdot 10^{-4}\}$. With 100 simulations per parameter combination, every panel shows 2500 simulations.

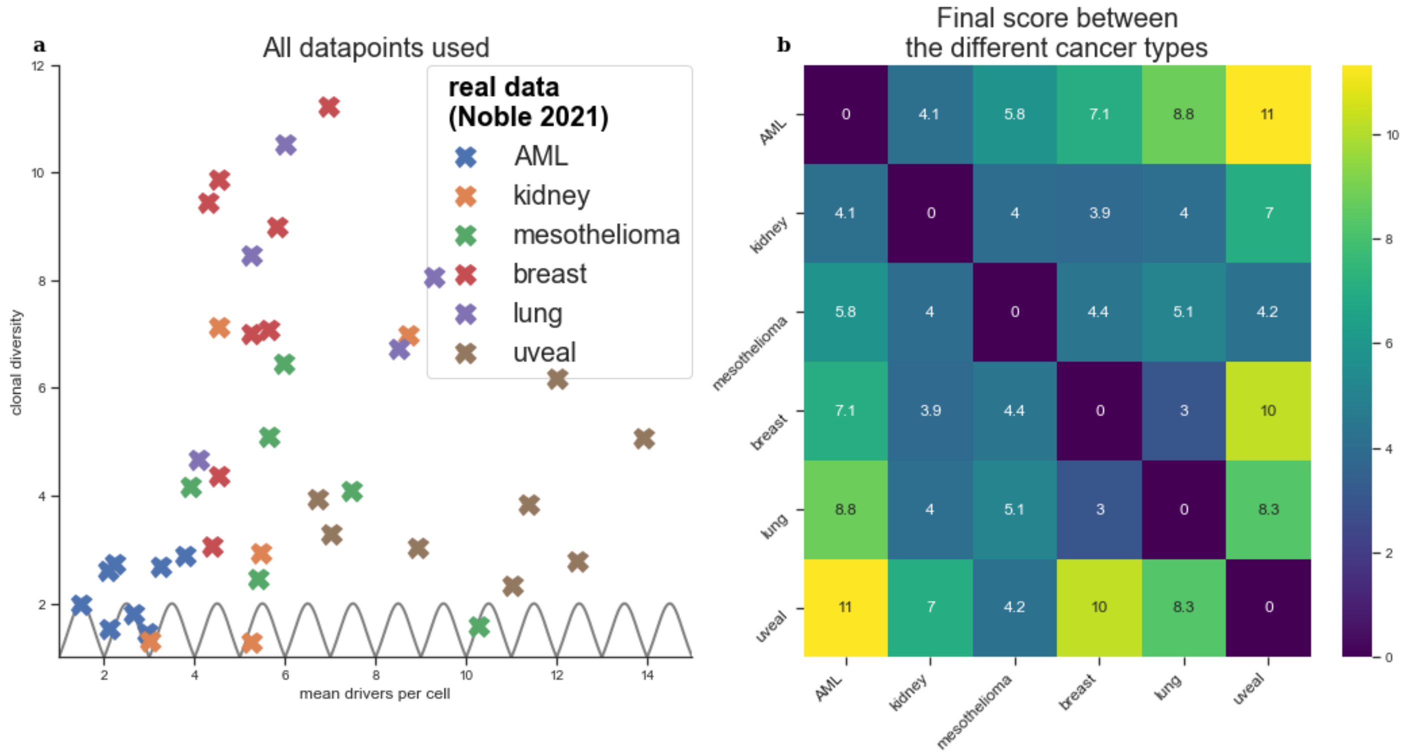

Supplementary Figure 12: a) All tumour samples used in the manuscript for the 6 datasets. b) Final score **S** (defined as the mean shortest distance for each datapoint, Eq. 22) between the 6 datasets.

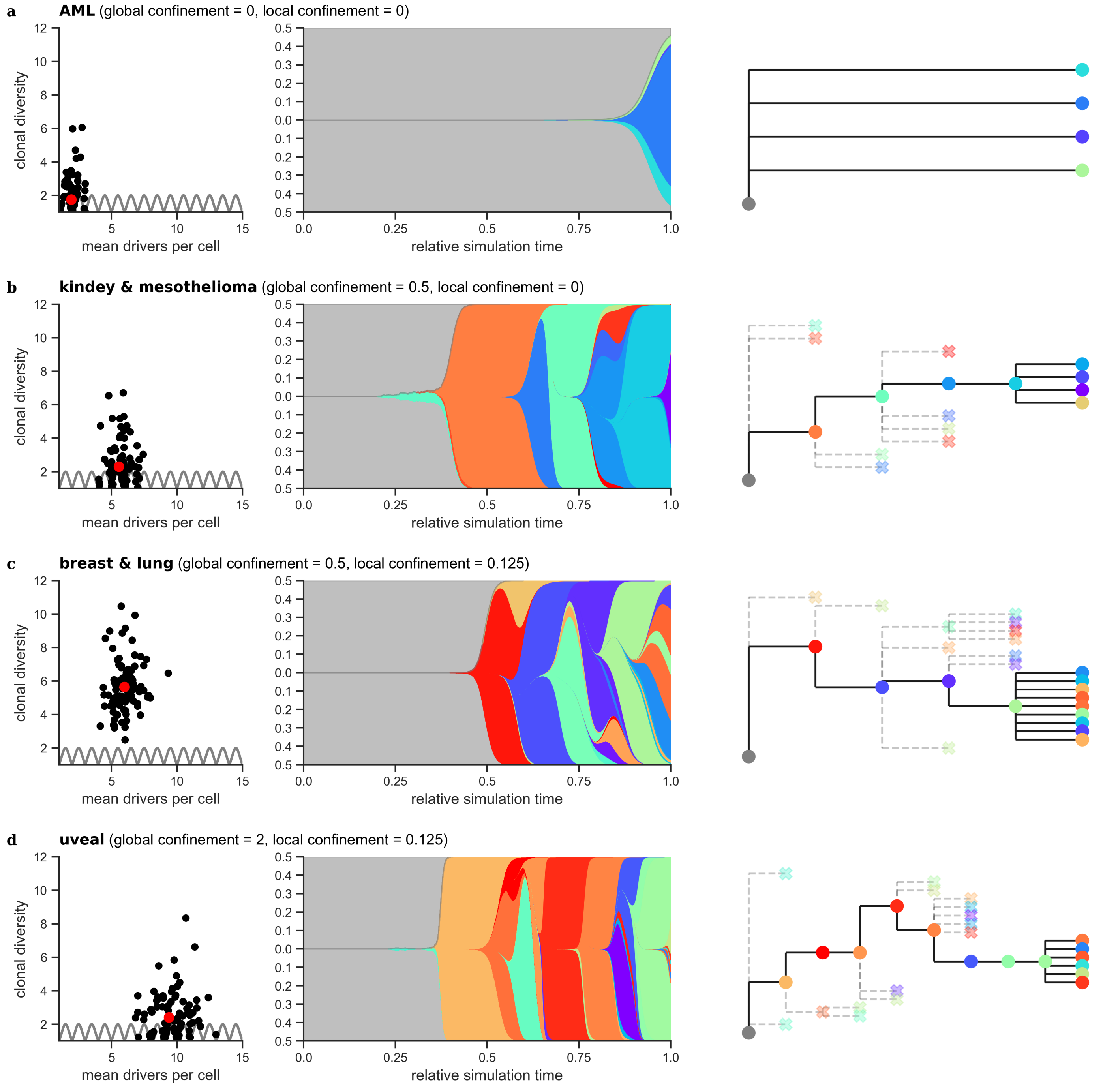

Supplementary Figure 13: Representative evolutionary patterns for the global and local confinement combinations that best fit the 6 cancer datasets. Left columns show the distribution of the mean number of drivers per cell and the clonal diversity for all 100 simulations per parameterisation. The theoretical limit for clonal sweeps is shown as a grey line. The middle and right column show the clonal evolution of a representative simulation run, marked by a red circle, through a fish plot and a phylogenetic tree. For the phylogenetic trees, extinct clones (fewer than 1.000 alive cells) are crossed out. Note that for both the fish plots and phylogenetic trees we selected only clones with a population fraction higher than 1

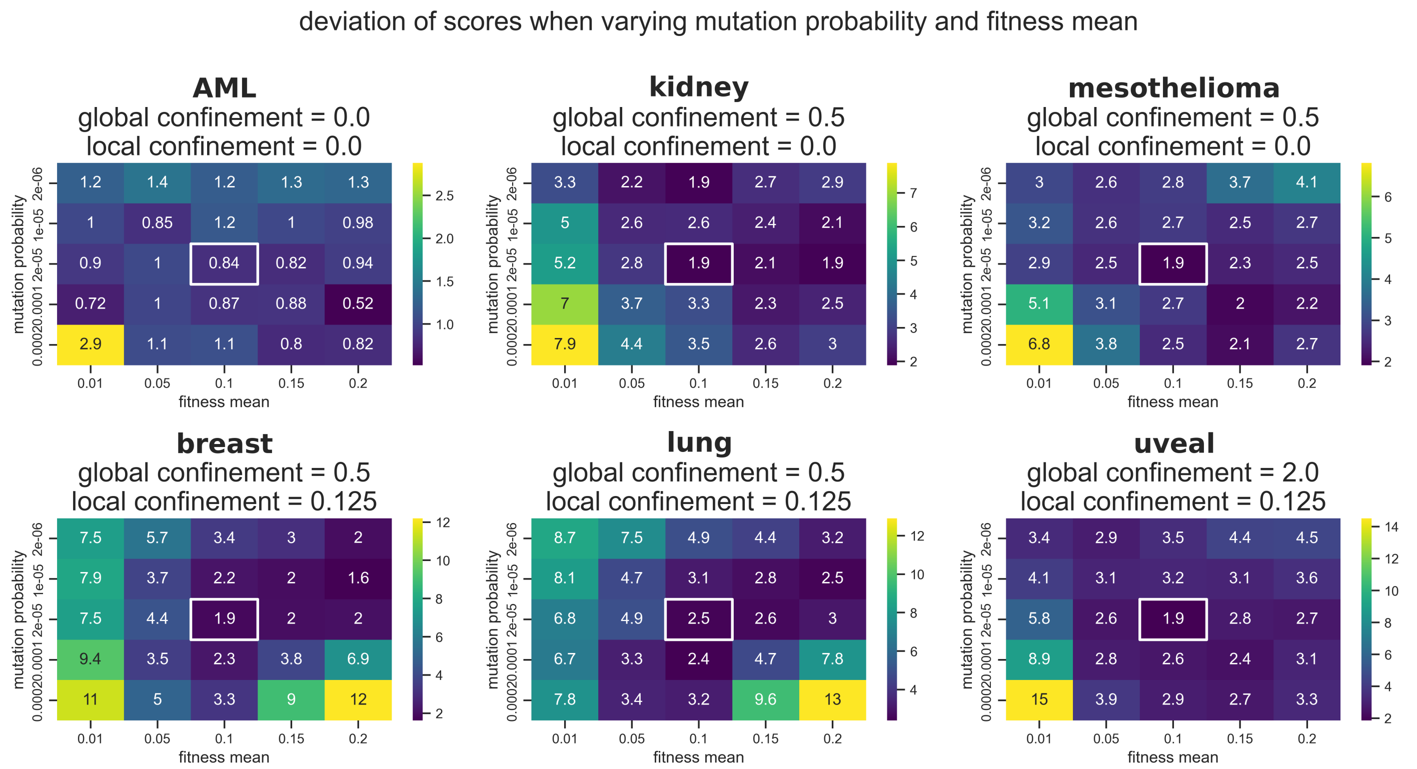

Supplementary Figure 14: For every cancer type, we tested the effect of varying the mutation probability and the mean fitness gain per driver on the goodness of fit. The two parameters were varied in the ranges $\theta_{\mathrm{fit}} \in \{0.01, 0.05, 0.1, 0.15, 0.2\}$ and $\theta_{\mathrm{mut}} \in \{2 \cdot 10^{-6}, 10^{-5}, 2 \cdot 10^{-5}, 10^{-4}, 2 \cdot 10^{-4}\}$. The score was calculated as described in Sec. 2.9 and Sec. 3.2. Small variations in the fitness mean and mutation probability produced similarly good fits to the real datasets.

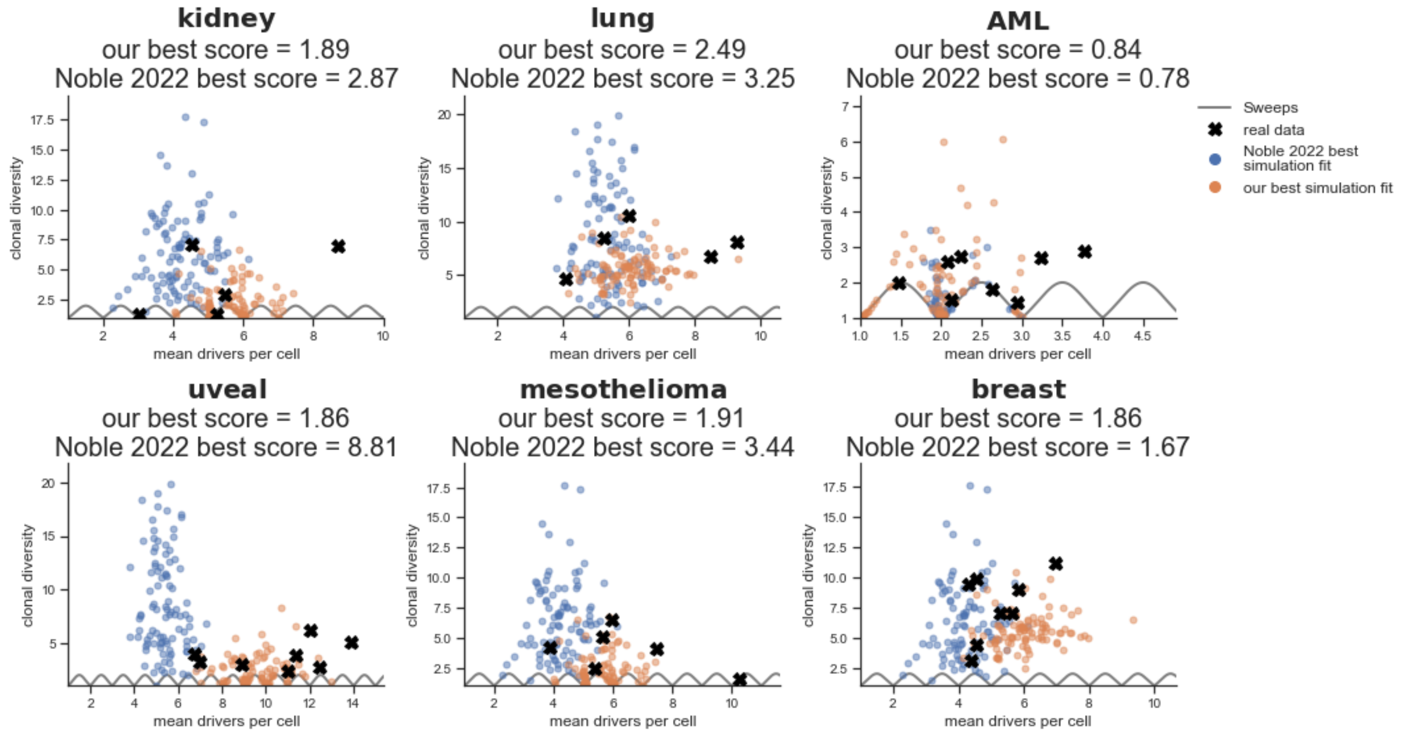

Supplementary Figure 15: Comparison of the best fit to the real data for both our simulation as well as the simulation from [Noble et al., 2022].

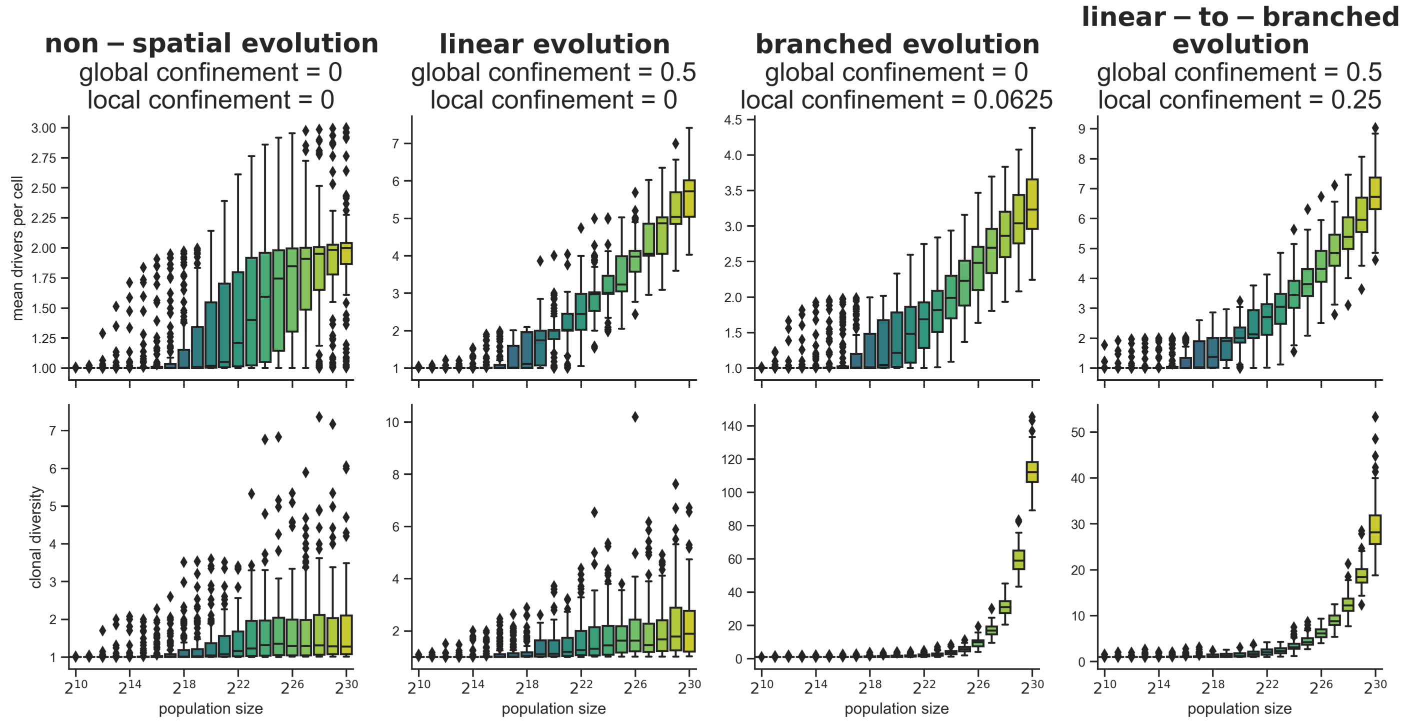

Supplementary Figure 16: The mean number of drivers per cell and the clonal diversity were evaluated for different time points of the tumour evolution. Note that the x-axes are log-scaled with base 2 and therefore every step on the x-axis corresponds to the doubling of the population which is motivated by the observation that across cancer types the doubling time remains roughly constant [Talkington and Durrett, 2015].

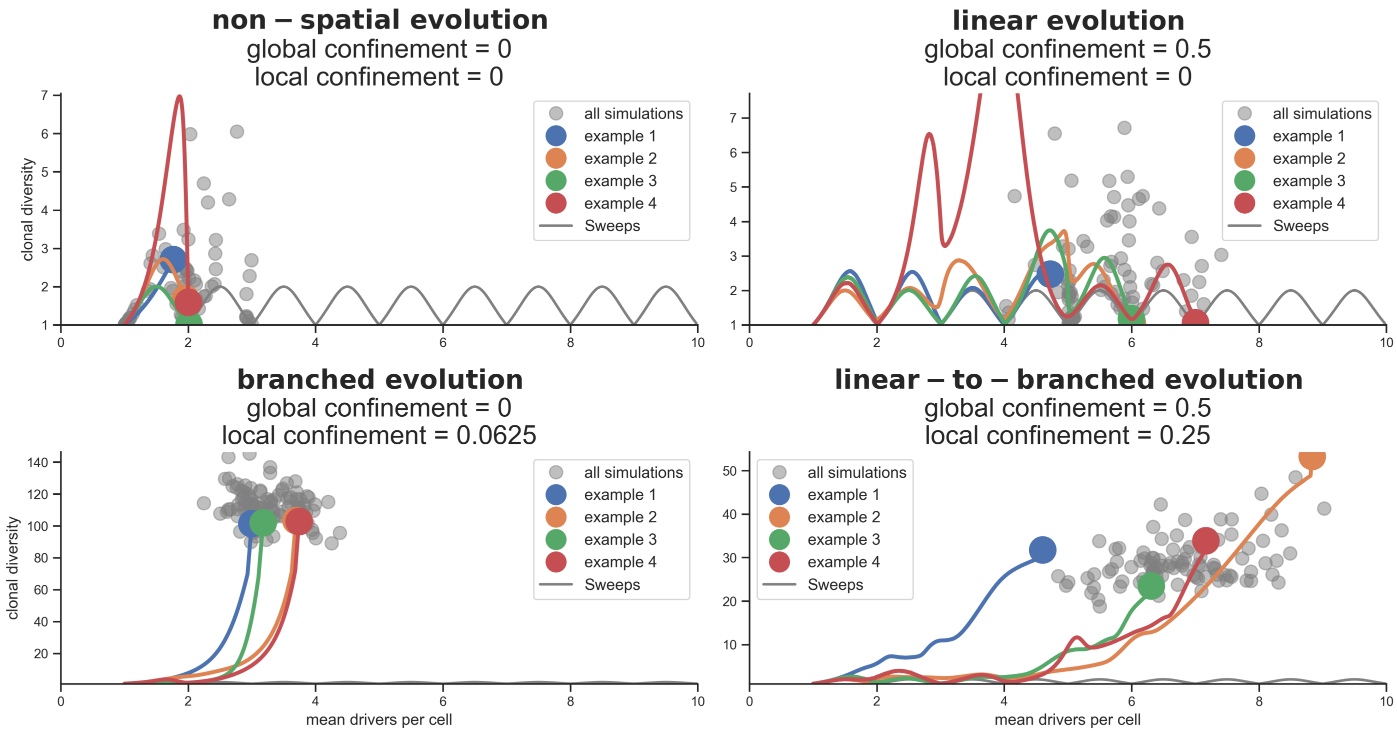

Supplementary Figure 17: For all four modes of evolution we show the trajectories of four exemplary simulations. Without any confinement, the trajectories were generally quite similar, as there are not external pressures on the growth. For the purely branching evolution, the trajectories were also quite similar as all of them continuously rose towards higher diversity and mainly differ in their slope. With global confinement, we start observing sequential clonal sweeps, however while some trajectories demonstrate a sweep after every new driver, some can remain heterogeneous for long periods, accumulating many drivers before a sweep. The sweeps can be dampened after a certain size is reached by addition of the local confinement, where the trajectories become the most heterogeneous, not displaying any shared behaviour.

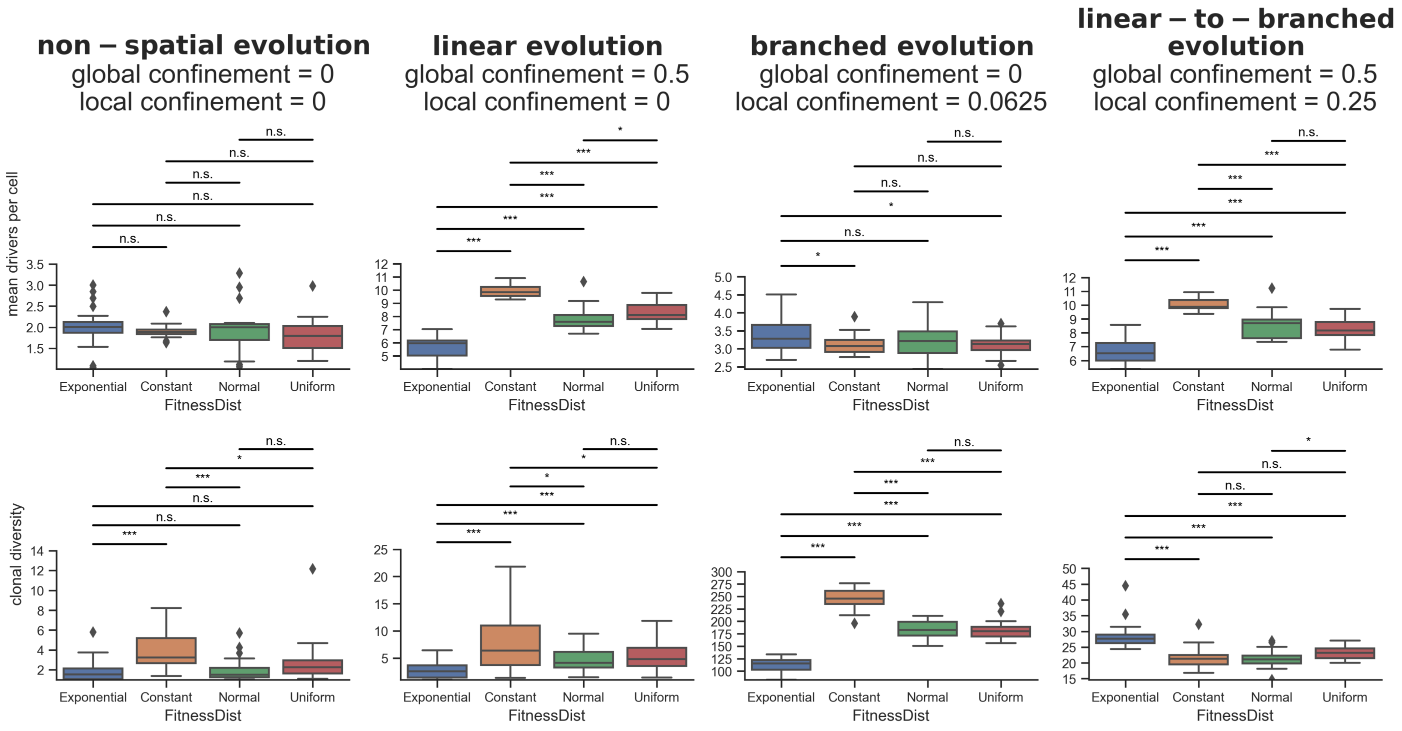

Supplementary Figure 18: Full effect of the choice of the fitness distribution as detailed in Sec. 3.4. of the main text. *: $p < 0.05$, **: $p < 0.005$, ***: $p < 0.0005$, n.s.: not significant.

References

Robert Noble, Dominik Burri, Cécile Le Sueur, Jeanne Lemant, Yannick Viossat, Jakob Nikolas Kather, and Niko Beerenwinkel. Spatial structure governs the mode of tumour evolution. *Nature Ecology & Evolution*, 6(2):207–217, February 2022. ISSN 2397-334X. doi: 10.1038/s41559-021-01615-9. URL `https://www.nature.com/articles/s41559-021-01615-9`.

Anne Talkington and Rick Durrett. Estimating tumor growth rates in vivo. *Bulletin of mathematical biology*, 77(10):1934–1954, October 2015. ISSN 0092-8240. doi: 10.1007/s11538-015-0110-8. URL `https://www.ncbi.nlm.nih.gov/pmc/articles/PMC4764475/`.
